# Multi-scale integrative analyses identify THBS2^+^ cancer-associated fibroblasts as a key orchestrator promoting aggressiveness in early-stage lung adenocarcinoma

**DOI:** 10.7150/thno.69590

**Published:** 2022-03-28

**Authors:** Haitang Yang, Beibei Sun, Liwen Fan, Wenyan Ma, Ke Xu, Sean R. R. Hall, Zhexin Wang, Ralph A. Schmid, Ren-Wang Peng, Thomas M. Marti, Wen Gao, Jianlin Xu, Weiwei Yang, Feng Yao

**Affiliations:** 1 Department of Thoracic Surgery, Shanghai Chest Hospital, Shanghai Jiao Tong University; Shanghai, 200030, People's Republic of China.; 2 Institute for Thoracic Oncology, Shanghai Chest Hospital, Shanghai Jiao Tong University; Shanghai, 200030, People's Republic of China.; 3 Clinical Research Center, Shanghai Chest Hospital, Shanghai Jiao Tong University, Shanghai, 200030, People's Republic of China.; 4 Wyss Institute for Biologically Inspired Engineering, Harvard University; United States.; 5 Division of General Thoracic Surgery, Department of BioMedical Research (DBMR), Inselspital, Bern University Hospital, University of Bern; Bern, 3010, Switzerland.; 6 Department of Thoracic Surgery, Huadong Hospital Affiliated to FuDan University, China.; 7 Department of Respiratory Medicine, Shanghai Chest Hospital, Jiao Tong University; Shanghai, 200030, People's Republic of China.; 8 School of Life Science, Hangzhou Institute for Advanced Study, University of Chinese Academy of Sciences, Hangzhou 310024, People's Republic of China.

**Keywords:** early-stage lung adenocarcinoma, THBS2, exosome, cancer-associated fibroblast, immunotherapy

## Abstract

**Rationale:** Subsets of patients with early-stage lung adenocarcinoma (LUAD) have a poor post-surgical course after curative surgery. However, biomarkers stratifying this high-risk subset and molecular underpinnings underlying the aggressive phenotype remain unclear.

**Methods:** We integrated bulk and single-cell transcriptomics, proteomics, secretome and spatial profiling of clinical early-stage LUAD samples to identify molecular underpinnings that promote the aggressive phenotype.

**Results:** We identified and validated THBS2, at multi-omic levels, as a tumor size-independent biomarker that robustly predicted post-surgical survival in multiple independent clinical cohorts of early-stage LUAD. Furthermore, scRNA-seq data revealed that THBS2 is exclusively derived from a specific cancer-associated fibroblast (CAF) subset that is distinct from CAFs defined by classical markers. Interestingly, our data demonstrated that THBS2 was preferentially secreted via exosomes in early-stage LUAD tumors with high aggressiveness, and its levels in the peripheral plasma associated with short recurrence-free survival. Further characterization showed that THBS2-high early-stage LUAD was characterized by suppressed antitumor immunity. Specifically, beyond tumor cells, THBS2+ CAFs mainly interact with B and CD8+ T lymphocytes as well as macrophages within tumor microenvironment of early-stage LUAD, and THBS2-high LUAD was associated with decreased immune cell infiltrates but increased immune exhaustion marker. Clinically, high THBS2 expression predicted poor response to immunotherapies and short post-treatment survival of patients. Finally, THBS2 recombinant protein suppressed *ex vivo* T cells proliferation and promoted *in vivo* LUAD tumor growth and distant micro-metastasis.

**Conclusions:** Our multi-level analyses uncovered tumor-specific THBS2+ CAFs as a key orchestrator promoting aggressiveness in early-stage LUAD.

## Introduction

Adenocarcinoma is the most common histological subtype of lung cancer. The lung cancer screening program greatly facilitates the early detection of lung adenocarcinoma (LUAD), leading to an improved prognosis over the years 1.

For early-stage LUAD patients without lymph node (N) metastasis (pathological [p] N0 stage), surgical resection can achieve a high curative rate, and adjuvant therapies are generally not recommended afterward. However, up to 20%∼30% of these early-stage patients have a poor prognosis with a significantly compromised 5-year overall survival (OS) and recurrence-free survival (RFS) 2, defining a high-risk subset who may need additional adjuvant therapies after curative surgery.

In view of the presence of high-risk subsets, it is crucial to identify specific biomarkers that can stratify pN0-stage LUAD patients for subsequent precise management and facilitate the development of novel targeted strategies. To achieve this, extensive studies have been conducted to uncover potential classification models. Currently, a well-established and widely used model is the new IASLC (International Association for the Study of Lung Cancer)/ATS (the American Thoracic Society)/ERS (the European Respiratory Society) classification system 3, which displays significant prognostic and predictive values regarding tumor recurrence and death 4. According to this model, early-stage LUADs predominant with mucinous, colloid, solid, or micropapillary components are characterized by dismal prognosis, thus defining a histological subtype-based model to identify high-risk subsets. Apart from that, previous studies also demonstrated other predictive clinicopathological biomarkers to determine the high-risk subsets 5, 6, e.g., tumor size, well-/moderately-/poorly differentiated histological status, visceral pleural involvement, lymphovascular invasion, despite the presence of heterogeneous conclusions 7. However, the majority of these predictive models are histology- and phenotype-based only, but lack the underlying molecular underpinnings that drive this aggressive subtype. Given the absence of molecular characterization, it is still unknown about the optimal management for this high-risk subset, in that it remains controversial whether conventional adjuvant chemotherapy could generate benefits 8-11.

Besides, the above models are highly restricted to the availability of tumor tissue, which involves invasive procedures and difficulties to obtain serial samples. By contrast, liquid biopsy, e.g. cell-free DNA (cfDNA), circulating tumor cells (CTCs) and plasma exosomes, can overcome these disadvantages 12. Among these, exosomes represent a more interesting liquid biomarker from the perspective of pathobiological roles in tumors 13. An increasing number of studies have revealed that exosomes, which are ~40 to 100 nm vesicles secreted by a wide range of cell types, mediate the cross-talk among cells within or outside the tumor microenvironment (TME), promote cancer drug resistance and create a metastatic niche to facilitate tumor recurrence/metastasis 14.

Accumulating evidence has revealed the critical role of TME in promoting disease progression and treatment resistance 15. The TME comprises various cell types, including stromal cells (predominantly cancer-associated fibroblasts (CAFs)), immune cells, and extracellular matrix (ECM) components. Among those, CAFs are the most abundant cell type in a variety of carcinomas, mediating the production of ECM components, such as collagens, glycosaminoglycans and glycoproteins, enhancing the invasive activity of cancer cells, and suppressing anti-tumor immunity 16. Particularly, CAFs also represent one of the major sources secreting exosomes in cancer 17. Nevertheless, the role of TME in the progression of early-stage LUAD is still unclear.

Here, by integrating multi-omic analyses of independent datasets, we provided the first evidence that THBS2, an exosome protein secreted by a specific subset of CAFs, serves as a robust biomarker for predicting OS and RFS as well as clinical treatment response in patients with pN0-stage LUAD. Functionally, CAFs-derived THBS2 promoted the aggressive phenotype by presumably modulating both cancer cells and tumor immunity, identifying THBS2+ CAFs as a candidate therapeutic target in early-stage LUAD.

## Methods

### Study design

**Figure 1:**
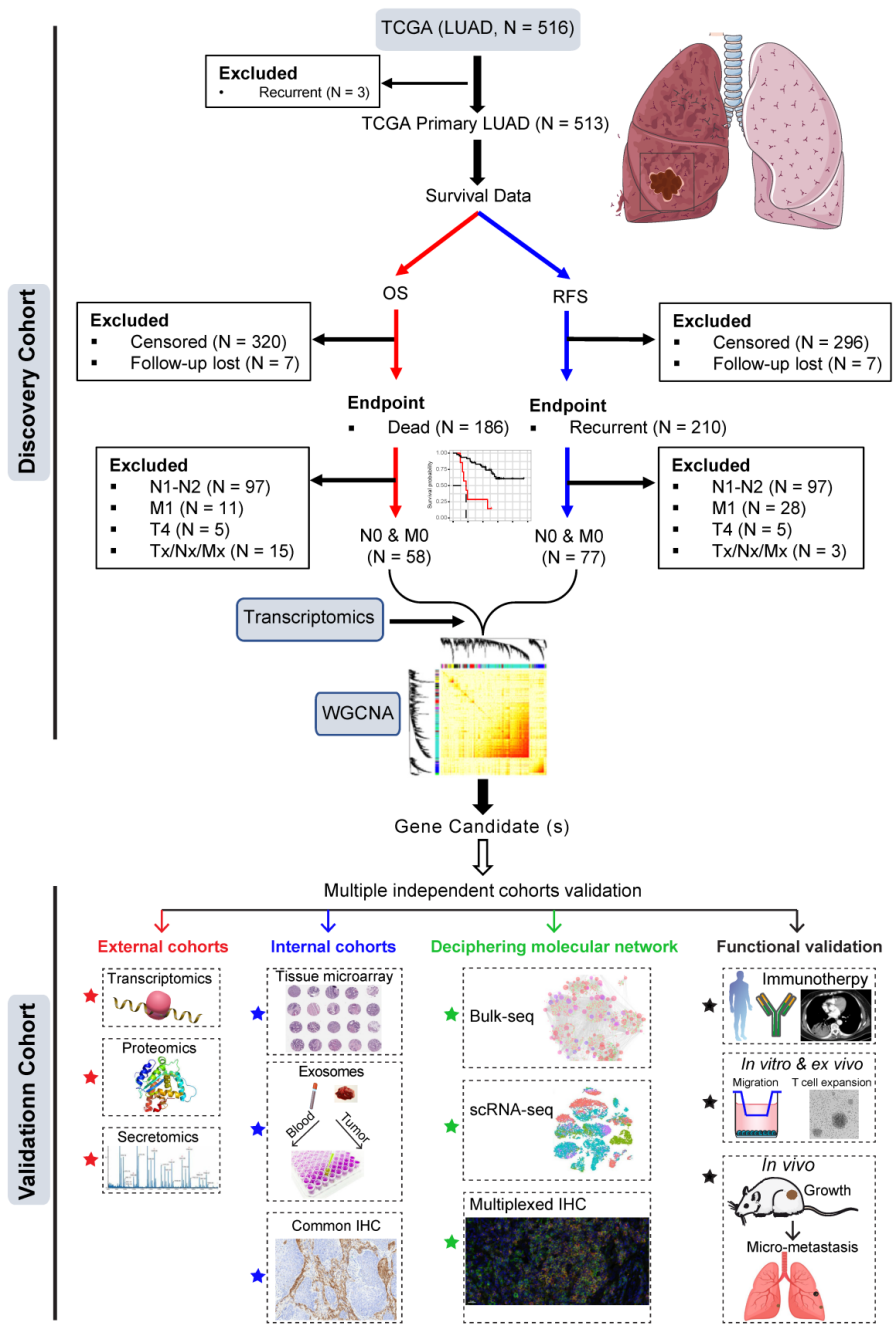
** Study design.** Early pN0-stage lung adenocarcinoma (LUAD) with complete survival endpoints (recurrence-free survival (RFS) and overall survival (OS) from The Cancer Genome Atlas (TCGA) database) was used as a training cohort. Following this, WGCNA (weighted gene co-expression analysis) analysis is used to identify molecular clusters that correlate with RFS and OS, which were then validated using multiple external and internal resected pN0-stage LUAD cohorts. Finally, the potential molecular networks and biological functions of the candidate molecules were deciphered with multi-dimensional evidence.

The training dataset was based on The Cancer Genome Atlas (TCGA) LUAD cohort (**Figure 1**). Patients who had primary LUAD at pathologically confirmed pN0/M0-stage after radical surgery and reached the endpoints (cancer-related death or tumor recurrence) were included. Weighted gene co-expression analysis (WGCNA) was then performed by correlating the transcriptomic data (treatment-naïve) with clinical survival data (cancer-specific OS and RFS).

The most negatively correlated cluster (module) with RFS and OS was then selected to identify potential gene candidates whose high expression predicts poor prognosis of patients with early pN0-stage LUAD. Subsequently, the prognostic capacity of the selected gene candidates was evaluated in the training cohort and independent external (at the transcriptomic and proteomic levels) and internal (at the protein level) cohorts. Finally, single-cell transcriptome and multiplexed immunohistochemistry staining analyses, as well as *in vitro* and *in vivo* functional experiments were performed to decipher the major cellular subsets that contribute to the aggressive phenotype in pN0-stage LUAD (**Figure 1**).

### WGCNA and Function Enrichment Analyses

To identify the gene expression profiles associated with the protein levels of RFS and OS in pN0-stage LUAD, we applied the R package “WGCNA” to whole-genome transcriptomic data. In WGCNA, genes were clustered according to their co-expression patterns 18-20, with which a gene co-expression network was then constructed. Genes were grouped into different modules (clusters) using the dynamic tree cut algorithm, according to topological overlap matrix (TOM)-based dissimilarity. The module eigengene (ME) was calculated based on the first principal component of each module. The ME values were correlated (Pearson) with sample traits (RFS and OS). Here, we set the soft-thresholding power at 12 (scale-free R2 was approximately 0.9), cut height at 0.25, and minimal module size to 30 to identify key modules. The module significantly correlated with sample traits was selected to explore its biological functions, such as Gene Ontology (GO), Kyoto Encyclopedia of Genes and Genomes (KEGG) and Reactome pathway enrichment analyses, using the R package “clusterprofiler” 21, 22. Hub genes were defined as the top 30 intramodular connected genes.

### Patient samples (tissue and blood)

The treatment-naïve samples of lung cancer patients for the present study were all taken from hospitalized patients in the Department of Thoracic Surgery at Shanghai Chest Hospital.

Peripheral blood samples were collected from treatment-naïve LUAD patients and healthy donors. Samples were centrifuged at 4 °C for 10 min at 1000 g to separate plasma from blood cells. Supernatants were collected, divided into aliquots and stored at -80 °C until use.

This study was approved by the institutional review board (#KS(Y)21316). All patients had signed informed consent for inclusion of their clinical data and specimens in our Lung Biobank and use in research projects, according to the recommendation of the ethical committee of Shanghai Chest Hospital.

### Follow-up

RFS was defined as the interval between surgery and recurrence; if recurrence was not diagnosed, the date of death or last follow-up was recorded. OS was defined as the interval between surgery and death.

### Immunotherapy response assessments

After completion of two cycles of immunotherapy, positron emission tomography/computed tomography (PET/CT) or CT scans were performed to evaluate the therapeutic response. The response was assessed based on the Response Evaluation Criteria in Solid Tumors (RECIST) (version 1.1) 23.

### Preparation of single-cell suspensions from surgically resected samples

#### Sample preparation

Two paired (primary lung tumor [pT1N0M0] and matched normal lung tissue) lung samples were collected and immediately transferred for single-cell isolation. Tumor tissues and tumor-adjacent normal lung tissues were obtained during surgery at Huadong Hospital (Shanghai, China). Tumor samples were collected from three different sites of the tumor bed. Normal lung tissue was separated from the malignant region by at least 5 cm. The tissues were rinsed with cold PBS (Cytiva, SH30256.01) to wash out the blood and dead cells. Then, they were put in 1.5 mL cooled tissue storage solution (Miltenyi, 130-100-008) and transferred to the laboratory on ice. The tissues were minced into small pieces (smaller than 1 mm^3^) within 5 min, subjected to digestion buffer (4.7 mL DMEM (Cytiva, SH30243.01) + 325 μL enzyme mix (Miltenyi, 130-095-929)) and incubated at 37 ℃ for 30 min on a shaker. The samples were passed through a 70 μm filter (Miltenyi, 130-095-823) and centrifuged at 300 g at 4 ℃ for 7 min. The remaining blood cells and the dead cells were removed using red blood cell lysis solution (Miltenyi, 130-094-183) and Ficoll-Paque PLUS (Cytiva, 17144002-1) separation. The cell pellets were finally resuspended in DMEM + 10% FBS (Thermo Fisher Scientific, 10099141C) before microscopic inspection and scRNA-seq library construction.

#### Single-cell RNA library construction, sequencing and data processing

Single-cell suspensions (1 × 10^6^/mL), of which the viability was higher than 80%, were submitted to 10X genomics Chromium Controller to generate single-cell gel beads in emulsion (GEMs). The library was constructed by Chromium Next GEM Chip G Single Cell Kit, Single Cell 3' GEM, Library & Gel Bead Kit v3.1, Library Construction Kit v3.1, and i7 Multiplex Kit (10X genomics) following the manufacturer's instruction. Briefly, the captured cells were lysed, and the released RNA was barcoded via reverse transcription in individual GEMs. Reverse transcription was performed at 53 °C for 45 min, followed by 85 °C for 5 min, after which the temperature was held at 4 °C. Complementary DNA amplification and quantification were performed to build the 3' gene libraries, whose quality was assessed by a 2100 Bioanalyzer (Agilent). Then, libraries were sequenced by Illumina Nova-seq 6000 with a paired-end 150-base pair (PE150) reading strategy.

#### scRNA-seq data processing

Raw gene expression matrix was generated for each sample using the 10X genomics Cell Ranger toolkit (version 4.0.0) and mapped to the GRCh38 human reference genome. After that, the output was analyzed by R software (version 3.6.3) with the Seurat package (version 3.2.0) for quality control and downstream analysis 24. In brief, genes expressed at a proportion >0.1% of the data were selected for further analyses. Low-quality cells were removed if they met the following criteria: (1) < 500 unique molecular identifiers (UMIs), (2) < 200 or > 10000 genes, (3) > 10% unique molecular identifiers (UMIs) derived from the mitochondrial genome, (4) > 10% UMIs derived from the red-cell genome, or (5) predicted doublets by the Scrublet package (version 3.7.3) 25.

#### scRNA-seq data normalization, dimensional reduction, and clustering

After the removal of low-quality cells, the datasets collected from different samples were integrated using FindIntegrationAnchors and FindIntegrateData function with default parameters to remove the batch effect 26. The gene expression matrix was normalized by the NormalizeData function, and 2000 features with high cell-to-cell variation were calculated using the FindVariableFeatures function. To reduce the dimensionality of the datasets, the RunPCA function was conducted with default parameters on scaled data generated by the ScaleData function. During this process, we implemented Clustree algorithm (“clustree” R package; https://cran.r-project.org/web/packages/clustree/vignettes/clustree.html) to increase gene representation and achieve optimal cluster separation 27. Next, the ElbowPlot, DimHeatmap, and JackStrawPlot functions were used to identify the true dimensionality of each dataset, as recommended by the Seurat developers. Last, the first 20 principal components were applied to clustering by FindNeighbors and FindClusters functions, and RunTSNE function was performed with default settings for nonlinear dimensional reduction. For all 43,779 cells, the clustering results were visualized with the UMAP (Uniform Manifold Approximation and Projection) scatter plot.

#### Cell type annotation and cluster marker identification

The annotations of cell identity on each cluster were defined by the expression of known marker genes, which were identified using the FindAllMarkers function provided by Seurat. The fibroblasts were identified according to the known markers: *DCN*, *LUM*, *COL1A1*, *COL1A2*. Clusters were also confirmed by identifying significantly highly expressed marker genes in each cluster and then comparing them with the known cell-type-specific marker genes. By implementing the Clustree algorithm (“clustree” R package), the 332 CAFs were further categorized into 10 clusters.

#### Gene set enrichment analysis

We conducted the gene set enrichment analysis (GSEA) for differentially expressed genes between THBS2+ (positive) and THBS2- (negative) cancer-associated fibroblasts using clusterProfiler (version 4.0.5) and GSEABase (version 1.54.0) R toolkit with default settings 28. Gene set used was hallmark gene set from the Molecular Signatures Database (MSigDB) 29.

#### Cell-cell interaction network in lung cancer samples

Cell-cell interaction network was determined by the CellChat R toolkit (version 1.1.2, https://github.com/sqjin/CellChat), an intercellular interaction analysis tool that studies ligand-receptor action in specific signaling pathways 30. To explore the intercellular interaction in the tumor microenvironment, gene expression matrices and metadata with major cell annotations from cancer samples were used as input for the CellChat software. Briefly, the cell-cell interaction was measured by quantification of ligand-receptor pairs among different cell types.

### Transwell assay

The migration assay was performed using Transwell plates (24-well chamber with 8.0 µm pore size; Corning, NY, USA). Cells were starved overnight in media containing only 1% FBS. Briefly, 10^5^ LUAD cells (incubated with PBS or 50 ng/mL THBS2 recombinant protein) were resuspended in the upper compartment (containing 200 µL FBS-free medium), with the lower compartment supplied with 10% FBS-containing media (800 µL). All of the results were obtained from three independent experiments.

### Exosome isolation, purification and characterization

LUAD (pN0M0; N = 5) and normal adjacent tissue (NAT; N = 5), as well as blood plasma samples (2 mL; pN0M0-LUAD, N = 5; healthy controls, N = 5), were obtained freshly from Shanghai Chest Hospital. Combination of size-exclusion chromatography (SEC) (Echo9101A-30 mL, Exosupur kit, Echobiotech) and ultracentrifugation was used to purify exosomes in plasma 31 and tissue 32, 33.

For isolating exosomes from blood plasma, fresh peripheral blood samples from individuals were collected in EDTA tubes following a regular venipuncture procedure. After centrifugation at 3,000 ×g for 15 min at 4 °C, the plasma was aspirated and stored at -80 °C before use. samples were filtered through a 0.22 μm filter and added to the SEC column, 2 mL collected plasma fractions were centrifuged at 150,000 × g, 4 °C for 4 h to further pellet the exosomes 31. The pellet was resuspended in PBS and centrifuged again 150,000 × g, 4 °C for 2 h. Finally, the supernatant was removed and resuspended in 100 μL PBS. The quantities of isolated exosomes were determined using a BCA Protein Assay Kit (Beyotime, Shanghai, China). Concerning the comparison of exosomal THBS2 levels in the plasma of LUAD patients, we used the same volume (2 ml) of plasma (stored at -80 °C within 1 year).

For isolating exosomes from lung tissue, human pN0M0-stage LUAD and the normal adjacent lung tissues were gently dissociated into small pieces (~2 × 2 × 2 mm) and frozen at -80 °C. Exosomes were separated from tissue using the protocol established previously by Vella, et al. 32, with minor modifications. The dissociation mixture was based on the Miltenyi Human Tumor Dissociation Kit (Miltenyi Biotec, cat. no. 130-095-929). Before starting, enzymes H, R and A were resuspended according to the manufacturer's instructions. Dissociation mix containing 2.2 mL RPMI, 100 μL enzyme H, 50 μL enzyme R and 12.5 μL enzyme A was prepared immediately before use. A small (~200 mg) piece of tissue was weighed and briefly sliced on dry ice and then incubated in the dissociation mixture for 10-15 min at 37 °C. The dissociated tissue was filtered through a 70 μm filter gently twice to remove residual tissues. Then the suspension was spun at 300 × g for 10 min at 4 °C, and the supernatant was transferred to a fresh tube and spun at 2000 × g for 10 min at 4 °C. Cell-free supernatant was spun at 10,000 × g for 20 min at 4 °C and filtered through a 0.22 μm filter gently and slowly for further depletion of cell debris. The collected suspension was then processed by ultracentrifugation (UC) at 150,000 × g for 2 h at 4 °C. The pellet was resuspended in 1ml phosphate-buffered saline (PBS) and further purified using Exosupur® columns (Echobiotech, China). Fractions were concentrated to 200 μL by 100 kDa molecular weight cut-off Amicon® Ultra spin filters (Merck, Germany). Regarding the comparison of exosomal THBS2 levels in the LUAD tumor tissue, we used the same weight (0.5 g) of frozen (-80 °C) tumor tissue (removing the adjacent normal tissue).

The morphology of purified exosomes was identified by transmission electron microscopy (H-7650, Hitachi Ltd., Tokyo, Japan); briefly, 10 μL exosomes solution was placed on a copper mesh and incubated at room temperature for 1 min. After washing with sterile distilled water, the exosome was contrasted by uranyl acetate solution for 1 min. The sample was then dried for 2 min under incandescent light. The copper mesh was observed and photographed under a transmission electron microscope. The size and purity of exosomes were measured by Nanoparticle Tracking Analysis (NTA) using ZetaView PMX 110 Nanoparticle Analyzer (Particle Metrix, Meerbusch, Germany); the identity of exosomes was validated using three positive exosomal markers (CD9, TSG101 and HSP70) and one negative marker (Calnexin) by western blot.

### Exosome protein quality and enzyme-linked immunosorbent assay (ELISA)

#### Total Protein Extraction

The exosome samples isolated and purified at the same conditions were lysed with lysis buffer containing 100 mM NH4HCO3, 6M Urea and 0.2% SDS, followed by ultrasonication on ice. The lysate was centrifuged at 12, 000 g for 15 min at 4 ℃ and the supernatant was collected. Each sample was reduced with 10 mM DTT for 1 h at 56 ℃ and alkylated with iodoacetamide for 1 h at room temperature in the dark. Then samples were mixed with 4 times volume of acetone and incubated at -20 ℃ for 2 h. After centrifugation, the precipitation was collected and washed by cold acetone. The pellet was dissolved by 0.1 M TEAB and 6 M Urea.

#### Protein Quality Test

The protein quality of samples was calculated by the BSA standard protein solutions curve with PierceTM BCA Protein Assay Kit (Product No. 23,225, Thermo Scientific, USA). 10 μL standard samples were pipetted into 96-well plates, then added 200 μL BCA kit to each well. Then the plate was covered and incubated at 37 ℃ for 30 min. The absorbance was set at 562 nm on the plate reader, and the standard curve was used to measure the protein concentrations of each isolated sample. Each sample solution with different dilution multiples was repeated three times.

20 μL protein sample was loaded to 12% SDS-PAGE gel electrophoresis. The concentrated gel was performed at 80 V for 20 min, and the separation gel was performed at 120 V for 90 min. The gel was stained by coomassie brilliant blue R-250 and decolored until the bands were visualized clearly. assessed using a microplate reader (Bio-Rad Laboratories, Hercules, CA, USA).

#### Proteinase K Assay

To determine the location (membrane or inside of exosomes) of exosomal THBS2 protein, proteinase K assay was performed, as described previously 34. 20 mL plasma of 6 LUAD patients were used to purify exosomes (500 μL; concentration: 1062 ng/μL), and were incubated in (1) PBS (control), (2) 1.2 μg/mL Proteinase K (Promega, Catalog Number: V3021; in PBS) alone, (3) 0.5% Triton X-100, or (4) combined Proteinase K and Triton X-100. The samples were then subjected to immunoblot to test TSG-101 (exosomal intra-membrane protein; as positive control), CD9 (exosomal trans-membrane protein; as positive control), THBS2.

### Common IHC, multiplexed IHC, tissue microarray (TMA) and quantitative analysis

Immunohistochemistry (IHC) was performed on 6-μm thick sections as previously described 35. Antibodies for common or multiplexed IHC were listed in **Table S1**.

For multiplexed IHC, three groups were set. 1) multiplexed IHC of THBS2, FAP, S100A4 and αSMA was performed according to the manufacturer's instructions (Absin, 5-Color Multiple IHC Kit; #abs50013; 2) multiplexed IHC of THBS2, CD19, CD8, FOXP3, CD68 and CD56 were performed according to the manufacturer's instructions (Absin, 7-Color Multiple IHC Kit; #abs50015; 3) multiplexed IHC of CD4, CD8, CD19 and PD-1 was performed according to manufacturer's instruction (Absin, 5-Color Multiple IHC Kit; #abs50013), as previously described 36. Briefly, slide sections of formalin-fixed paraffin-embedded (FFPE) block were deparaffinized in xylene and rehydrated in ethanol. After microwave antigen retrieval in heated citric acid buffer (pH 6.0) for 10 mins, endogenous peroxidase activity was blocked by 3% H_2_O_2_ for 10 mins, and nonspecific binding sites were blocked by goat serum for 10 mins. Primary antibodies were incubated for 1 h in a humidified chamber at room temperature, followed by incubation with the corresponding secondary horseradish peroxidase-conjugated polymer. Visualization of each target was accomplished using fluorescein TSA Plus (1:100). Then, the slide was again placed in a heated citric acid buffer (pH 6.0) using microwave antigen retrieval to remove redundant antibodies before the next step. Finally, nuclei were subsequently visualized with DAPI (Absin Bioscience Inc., 5 µg/mL), and the sections were coverslipped using antifade mounting medium (Absin 1:50).

For TMA construction, we retrospectively analyzed data from 93 patients who underwent surgery for lung cancer between 2004 and 2009 at the Department of Thoracic Surgery, Shanghai Chest Hospital. TMA slides were constructed as previously described in a protocol by Fedor HL et al. 37.

### Image acquisition and data quantification

For common IHC and TMA sections, whole slide images were acquired using Grundium Ocus^®^ microscope scanners. The THBS2 staining intensities of cancer and stromal cells in the images of full tissue sections were automatically analyzed and quantified using QuPath open-source software (version 0.2.4), where the DAB channel intensity of THBS2 (membrane and cytoplasm OD value) was extracted for each section 38. For the classification of cancer cells, stromal cells, and necroptosis, multiple training regions representing typical morphologies of cancer and stromal cells as well as necroptotic regions are annotated first. Based on this, the unique parameters of each cell type were generated, which were then applied to the whole slide images.

For multiplexed IHC, slides were scanned and imaged using the Pannoramic MIDI® platform and were analyzed in batches using HALO® and R scripts for the quantification of positively stained cells as previously described 36. Consequently, we were able to quantify the positively-stained cells with one or combination markers. With this, we know how many cells are positive for single (e.g. THBS2+ only), double (e.g. THBS2+S100A4+), or triple (e.g. THBS2+S100A4+FAP+), quadruple (e.g. THBS2+S100A4+FAP+αSMA+) staining.

### Public Data acquisition and bioinformatic analysis

#### LUAD patient datasets

Transcriptomic and proteomic profiles as well as clinical parameters of primary LUAD patients from multiple public cohorts, including 9 microarray datasets (GSE10072 39; GSE32863 40; GSE63459 41; GSE68571 42; GSE72094 43; GSE30219 44; GSE29013 45; GSE14814 46; GSE121841 47) from GEO (Gene Expression Omnibus; https://www.ncbi.nlm.nih.gov/geo/), 1 RNA-sequencing dataset from a high-quality East Asian LUAD cohort 48, 3 transcriptomic and proteomic profiles from three recent publications in Cell 49-51, and 1 RNA-Seq dataset from TCGA, as well as a dataset from the KM plotter portal (https://kmplot.com/analysis/) 52. Only patients who met the following three criteria were included: i) detailed TNM (8th) staging information containing stage I, IA, IB or T1-3N0M0 53; ii) overall survival information incorporating follow-up time and vital status; and iii) appropriate sample size. Of note, of the 5 datasets (GSE10072; GSE30219; GSE32863; GSE63459; GSE68571), there were only transcriptomic data of early-stage LUAD and matched normal lung but without the survival data in GSE10072 and GSE32863. Although the survival data in GSE63459 were available, the cancer-related death only occurred in 5 patients in the entire cohort, leading to the fact that there are no sufficient endpoint events for analysis. For GSE68571, the survival data were provided, but our analysis (not shown) suggested a dramatical survival difference between the THBS2-high vs. THBS2-low pN0-stage LUAD, although the significance was not reached, which is largely due to the short follow-up in this study cohort and thus the median survival in the two subgroups was not reached. In addition, concerning the proteomic profiles from three recent publications in Cell 49-51, only one 51 but not the other two datasets 49, 50 provided the survival data. However, in the former dataset 51, there was no data of the matched normal lung tissue available. As such, we were only able to show the association between THBS2 protein level and survival with the former dataset, whereas comparing the difference in the THBS2 protein level between the pN0-stage LUAD and matched normal lung tissue with the latter two datasets. The clinical information could be found in Supplementary Table 1 of each publication. Raw CEL files, the corresponding chip platform and metadata were downloaded and normalized. The R packages “limma” and “edgeR” were used to normalize the data and identify the differential gene or protein expression, respectively 35.

#### Reverse phase protein array (RPPA) dataset

The level 4 RPPA data (normalized and batch effects removed) of LUAD patients were obtained from The Cancer Proteome Atlas (TCPA) 54. Normalization of RPPA data was processed as follows: 1) calculate the median of each protein across all samples; 2) subtract the median (from step 1) from values of each protein in all samples; 3) calculate the median of all proteins in each sample; 4) subtract the median (from step 3) from values of each sample. The detailed information could be found at the TCPA portal: tcpaportal.org/tcpa/faq.html.

#### Immunotherapy datasets

Datasets with immunotherapy response data were downloaded and reanalyzed through GSE135222 (human lung cancer 55), GSE78220 (human melanoma 56), and GSE63557 (mouse mesothelioma 57).

#### Gene signatures

##### Immune subtype models

C1-6 immune subtype models were generated according to a previously curated dataset 58. Briefly, C1 (wound healing) subtype had elevated expression of angiogenic genes and a high proliferation rate; C2 (IFN-g dominant) subtype had the highest M1/M2 macrophage polarization, a strong CD8 signal and, together with C6, the greatest TCR diversity, and also showed a high proliferation rate; C3 (inflammatory) subtype was characterized by elevated Th17 and Th1 genes and low to moderate tumor cell proliferation; C4 (lymphocyte depleted) subtype displayed a more prominent macrophage signature, with Th1 suppressed and a high M2 response; C5 (immunologically quiet) subtype exhibited the lowest lymphocyte and highest macrophage responses, dominated by M2 macrophages. The C6 (TGF-b dominant) subtype displayed the highest TGF-b signature and a high lymphocytic infiltrate with an even distribution of the type I and type II T cells.

##### TME subtype models

TME subtype models were established by integrating multiple immunotherapy-associated dataset collections. The curated annotation and transcriptomic data of different cohorts were downloaded directly from the supplementary files of the corresponding publication 59.

##### Immune infiltrates estimation

QuanTIseq, an algorithm that was specifically developed for RNA-sequencing data, was used to 60 estimate immune cell infiltrates. Briefly, normalized expression data (as transcripts per millions (TPM)) of pN0-stage LUAD tumors of TCGA cohort were used as inputs and then quantified via deconvolution the proportions of six different immune cell types (CD8+ T cells, non-regulatory CD4+ T cells, B cells, M1 macrophages, M2 macrophages natural killer [NK] cells) using the “quantiseqr” package in R.

### Tumor purity evaluation

We implemented the ABSOLUTE-algorithm-based estimation of tumor purity, which could be directly downloaded from the UCSC portal (https://xenabrowser.net/datapages/, TCGA LUAD dataset).

### T cell proliferation

T cell proliferation and activation assay were described previously 61, 62. Briefly, peripheral blood mononuclear cells (PBMCs) were isolated from patient donors using Ficoll-PaqueTM Plus (GE Healthcare Life Sciences) density gradient centrifugation. Then, purified CD3+ T cells were isolated from the PBMCs using the EasySep™ Human T Cell Isolation Kit (StemCell Technologies; catalog No. #17751) according to the manufacturer's instructions. Single cells were expanded using an expansion media (Immunocult, StemCell Technologies; catalog No. #10981) consisting of 10 ng/mL of recombinant human IL-2 (StemCell Technologies; catalog #78036.1) and anti-CD3/CD28 beads (StemCell Technologies; catalog No. #10971). Cells were grown in tissue culture-treated 12-well plates, fresh media changes were made every 3-4 days until cell colonies were evident. Then the same number of PBMCs were cultured in the expansion media containing 0, 100, 200 or 500 ng/mL THBS2, respectively, for 96h.

### Animal experiments

Female BALB/c nude and C57BL/6 mice (6 weeks old) purchased from JSJ-lab (Shanghai, China) were used for animal experiments with the human LUAD cell line (A549) and mouse LUAD cell line (Lewis LLc cells), respectively. For A549 or LLc xenografts, tumor cells 1:1 mixed with Matrigel (356231; Corning) were subcutaneously inoculated in the left and right flanks (10^5^ cells/injection). Mice were divided into 2 groups: 1) PBS group (N = 7) and 2) THBS2 (50 µg/mL, human recombinant THBS2, Catalog #1635-T2-050, Bio-Techne China Co. Ltd.; mouse recombinant THBS2, Catalog #ABIN3011848, Atlas antibodies, Germany) group (N = 5). Tumor size/volume was calculated by the formula: (D × d^2^)/2, where “D” refers to the long tumor diameter and “d” the short tumor diameter 35, 63.

### Statistical analyses

Data are presented as the mean ± s.d., with the indicated sample size (n) representing biological replicates. Comparisons between two groups were carried out using parametric Student's two-tailed unpaired t-test for normally distributed data. If data were not distributed normally, a nonparametric Wilcoxon rank-sum test (for unpaired) was used between the two groups. Comparisons among three groups were determined by one-way/two-way analysis of variance (ANOVA) and Bonferroni's multiple comparison test. Statistical significance was determined by using GraphPad Prism 8 or R software (version 4.0.3, http://www.r project.org). Survival analysis was performed using the “survminer” and “survival” R packages. Tumor samples within all datasets were divided into two groups based on the best-separation cut-off value of THBS2 (mRNA or protein level) to plot the Kaplan-Meier survival curves and perform multivariate Cox regression (forest plot) analysis to evaluate the risk significance of each variable for RFS and OS. p < 0.05 was considered statistically significant.

## Results

### Gene clusters correlated with survival of patients with pN0-stage LUAD

**Figure 2:**
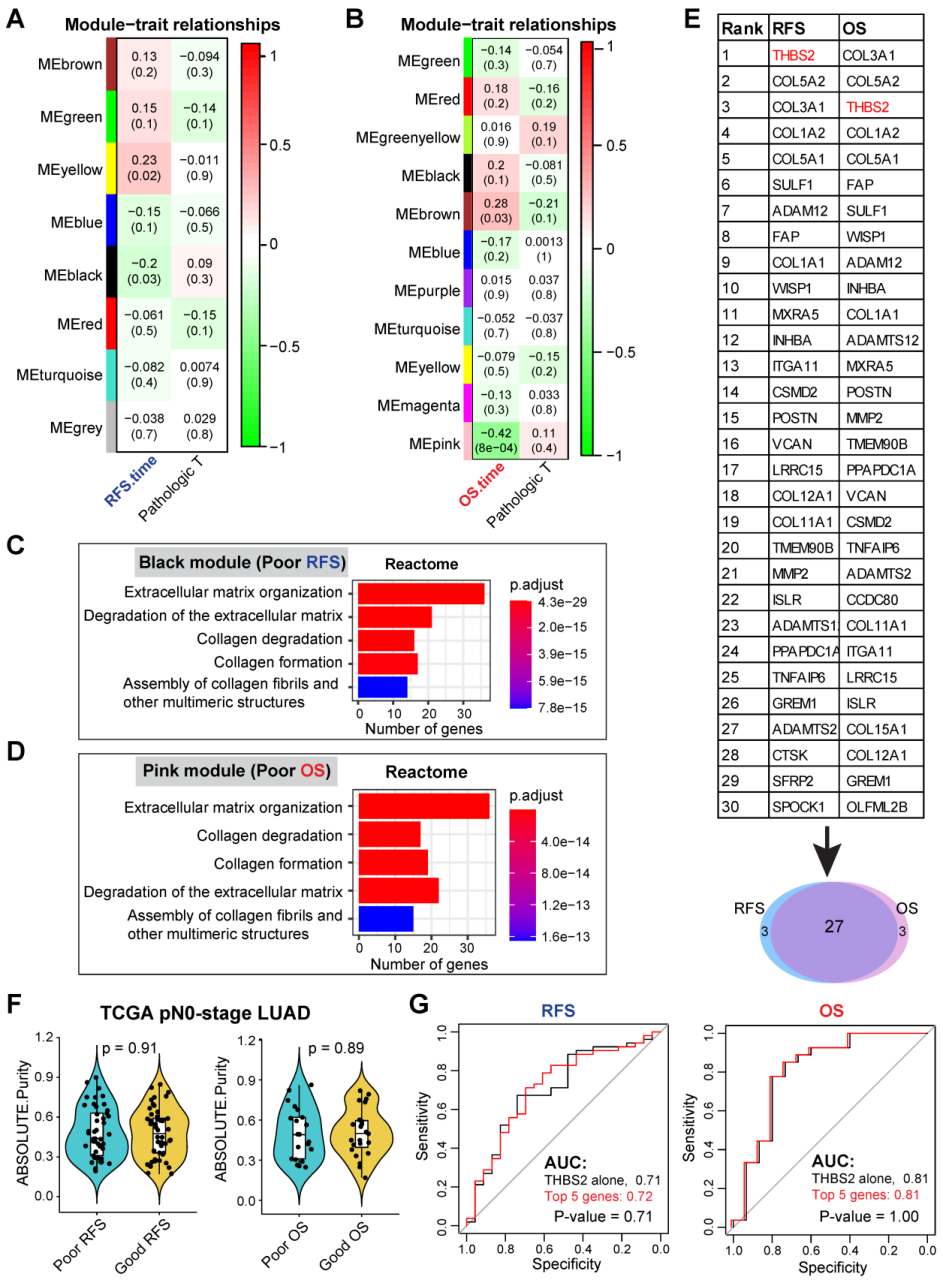
** WGCNA analysis identifies THBS2 as a candidate biomarker predictive of RFS and OS in early pN0-stage LUAD.** A, B, WGCNA analysis. Consensus network modules correlated with RFS and pathological (p) T-stage (tumor size) in the TCGA LUAD (lung adenocarcinoma) cohort using the eigenmodule (the first principal component of the module). Pearson correlation coefficient along with p-value in parentheses underneath; color-coded according to correlation coefficient (legend at right). The blue color indicates a negative correlation, while the red color represents a positive correlation. C, D, Reactome pathway enrichment analyses of genes in black (related to poor RFS; C) and pink (related to poor OS; D) modules. E, Top 30 connected genes in black (negatively correlated with RFS) and pink modules (negatively correlated with OS). Lower panel: Venn plot showing the overlap between RFS- and OS-related top 30 connected genes. F, The difference in tumor purity between pN0-LUAD patients with good and poor overall survival (OS). The ABSOLUTE-algorithm was used for the estimation of tumor purity, which was directly downloaded from the UCSC portal (https://xenabrowser.net/datapages/, TCGA LUAD dataset). P-value was calculated by two-sided student`s t-test. G, Comparison of the difference between the receiver operating characteristic (ROC) curves of two predictive models derived from THBS2 alone and the top 5 genes (THBS2, COL3A1, COL5A2, COL1A2, COL5A1), respectively.

Our discovery cohort revealed several gene modules that were significantly correlated with RFS (**Figure S1A; Figure 2A**) and OS (**Figure S1B; Figure 2B**) in pN0-stage LUAD. Genes in the positively correlated modules indicate that their abundance (co-expression) correlates with longer survival, while the negatively correlated modules signify that their abundance (co-expression) correlates with shorter survival. Notably, the correlation of these gene clusters with RFS and OS was independent of the pathological T (tumor size) stage (**Figure 2A, 2B**).

We then focused on the negatively correlated black (corresponding to RFS, p-value = 0.03) and pink (corresponding to OS, p-value = 8x10^-4^) modules, given that their high expression predicts poorer prognosis (**Figure 2A, 2B**), thus making them better biomarker candidates concerning clinical applications. GO biological functional analysis revealed that genes in the black (N = 168) and pink (N = 259) modules were mainly enriched for ECM/stromal signature (**Figure 2C, 2D; Figure S2A, 2B**).

Of note, there was a high overlap (27 out of 30) of the top 30 most connected genes between the black and pink modules (**Figure 2E**), thereby identifying the genes whose high expression was consistently related to tumor relapse and poor survival of patients with pN0-stage LUAD. Since the association of the ECM/stromal gene signature with prognosis could be due to the difference in stromal abundance between tumors from patients with poor and good prognosis, we then compared the tumor purity from the two groups (poor vs. good prognosis), and found out that there was no difference (**Figure 2F**). Collectively, we identified a set of genes whose expression correlated with post-surgical survival in patients with pN0-stage LUAD.

### Discovery of candidate biomarker protein THBS2

Among the top10 best-connected genes correlating with poor prognosis (both OS and RFS) (**Figure 2E**), integral membrane glycoprotein THBS2 (thrombospondin 2) was particularly interesting for the following reasons: 1) it is the top connected genes within RFS/OS (first/second-ranked); 2) it is a secreted glycoprotein that mediates cell-cell and cell-matrix interactions and plays a potentially critical role in cancer cell-stroma communications (**Figure S3A**); 3) like many other glycoproteins that have been routinely used as cancer diagnostic biomarkers in clinic, e.g. carcinoembryonic antigen and carbohydrate antigen 125, THBS2 can also be secreted by LUAD tumors (**Figure S3B**), and detected in peripheral blood of patients 64-66, highlighting its promises as a liquid biopsy marker for early-detection or tumor recurrence survilence of lung cancer; 4) More importantly, compared with THBS2 alone, combining the top 5 genes (*THBS2, COL3A1, COL5A2, COL1A2* and *COL5A1*) did not significantly improve the predictive ability for RFS and OS (**Figure 2G**), which is due to the highly mutual positive correlation among these genes (**Figure S3C**). This analysis highlights the rationale for using a single marker THBS2 instead of a combined gene-signature as a predictive biomarker; 5) Previous evidence has revealed THBS2 as a prognostic biomarker (either good or poor) in several cancer types 67-69; 6) However, little is known about its role in promoting the aggressiveness of early-stage LUAD. Together, these characteristics make THBS2 a highly interesting candidate from the perspectives of cancer detection and prognostic biomarkers, as well as a potential therapeutic target 70.

In the TCGA training cohort, separate univariate and multivariate Cox survival analyses confirmed that high expression of *THBS2* was associated with poor OS (**Figure S3D**) and RFS (**Figure S3E**) in pN0-stage LUAD. Additionally, we investigated the factors that potentially influence the expression of THBS2 in early-stage LUAD, and intriguingly, we identified that smoking history was significantly associated with THBS2 expression (**Table S2**), which requires further investigations. Moreover, compared to normal lung tissues, pN0-stage LUAD had a higher expression of *THBS2*, which was much higher in the recurrent LUAD samples (**Figure S3F**). Collectively, the above data suggest THBS2 as a potentially desired biomarker predicting the poor prognosis in pN0-stage LUAD.

### Cross-validation of THBS2 in multiple independent datasets

To evaluate the reliability of our findings, multiple independent, external and internal datasets were then included. Overall, clinical pN0-stage LUAD tumor samples had significantly higher expression of *THBS2* across all datasets compared to the matched normal lung tissue, despite the presence of its heterogeneous distribution among individual primary lung tumors (**Figure S4A**).

Mining multiple datasets revealed that high expression of *THBS2* was linked with poor OS and RFS in pN0-stage LUAD patients (**Figure S4B-D**). Recent real-world evidence supports the management of adjuvant chemotherapy in high-risk early-stage LUAD patients, despite the presence of heterogeneous conclusions 8-11. More recently, adjuvant epidermal growth factor receptor (EGFR) tyrosine kinase inhibitors (TKIs) were also recommended in IB-IIIA stage LUAD after surgery 71. In a recent resource dataset 48, we were able to evaluate the association between *THBS2* and the survival of pN0-stage LUAD patients treated with adjuvant chemotherapy or EGFR-TKIs after surgery. Interestingly, we observed that in pN0-stage I LUAD patients treated with chemotherapy or EGFR-TKIs, high expression of *THBS2* was also significantly associated with poor OS (**Figure S4B**), suggesting that THBS2 might mediate resistance to chemotherapy or EGFR-TKIs in LUAD patients. In agreement, analysis of an independent public RNA-seq dataset obtained from biopsies of *EGFR*-mutant LUAD treated with osimertinib (a third-generation of EGFR-TKIs) further confirmed that *THBS2* gene expression significantly increased in the post-osimertinib (resistance) biopsies (**Figure S4E**) 72. Previous evidence showed the failure of chemotherapy to provide additional survival benefits for patients with pN0-stage IB lung cancer after surgery 8, which was likely due to the absence of stratification biomarkers.

**Figure 3:**
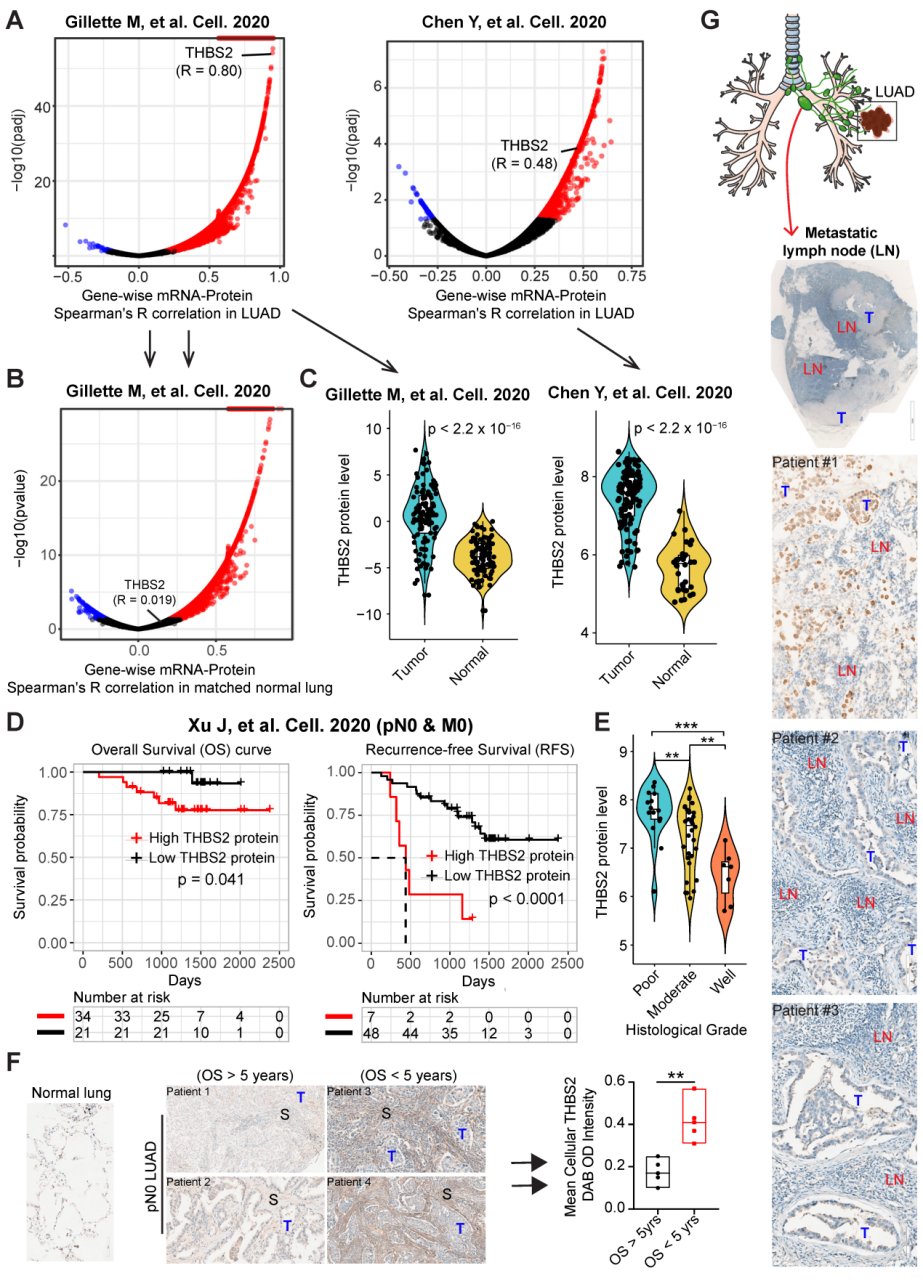
** Multiple external and internal validation of THBS2 as a prognostic biomarker.** A, B, Genome-wise mRNA-protein correlation (Spearman) analysis in lung adenocarcinoma (LUAD) tumors (A) and matched normal lung tissue (B). The blue color indicates a significantly negative correlation (adjusted p < 0.01), while the red color represents a significantly positive correlation. Data were downloaded and reanalyzed from Gillettle M, et al. Cell. 2020 and Chen Y, et al. Cell. 2020. C, Violin plots showing the protein level of THBS2 in pN0-stage LUAD compared with matched normal lung tissue. D, The association between THBS2 protein levels and OS and RFS in pN0-stage LUAD. Data were downloaded and reanalyzed from Xu J, et al. Cell. 2020. Of note, only one (Xu J, et al. Cell. 2020) but not the other two datasets (Gillette M, et al. Cell. 2020 and Chen Y, et al. Cell. 2020) provided the survival data. However, in the former dataset (Xu J, et al. Cell. 2020), there was no data of the matched normal lung tissue available. As such, we were only able to show the association of THBS2 protein level with survival with the former dataset, whereas comparing the difference in the THBS2 protein level between the pN0-stage LUAD and matched normal lung tissue with the later two datasets. The clinical information could be found in the Supplementary Table 1 of each publication. E, The association between THBS2 protein level and tumoral differentiation state in pN0-stage LUAD. Data were downloaded and reanalyzed from Xu J, et al. Cell. 2020. Of note, the differentiation stage of tumors is a critical histopathological classification of solid tumors, and is strongly associated with tumor behavior. Generally, tumors with poorer differentiation are more aggressive than their more differentiated counterparts. F, Internal immunohistochemistry (IHC) data showing the location of THBS2 expression in the samples from LUAD patients with short and long survival. **p < 0.01 by two-sided Welch`s t-test. Scale bar: 200 μm. G, Representative IHC showing the positive (upper panel: strong staining; middle/lower panel: moderate/weak staining) staining of THBS2 in three LUAD cases with regional lymph node metastasis. Scale bar: 100 μm.

Given that the high mRNA level of *THBS2* is predictive of prognosis, we next sought to determine whether there is a correlation between the mRNA and protein levels of THBS2 in LUAD samples. Based on two recent high-quality multi-omics datasets that represent Western and Asian LUAD populations 49, 50, we observed that there was a high consistency between the mRNA and protein levels of THBS2 in both Western and Asian LUAD cohorts (**Figure 3A**). Interestingly, this correlation does not exist in the matched normal lung tissue (**Figure 3B**), suggesting a LUAD-specific pattern. Along the same lines, at the protein level, pN0-stage I LUAD had significantly higher THBS2 protein expression than matched normal lung tissue (**Figure 3C**). Importantly, the high THBS2 protein level was linked with short OS and RFS in pN0-stage LUAD (**Figure 3D**) and poor differentiation status (marking a more aggressive state) of early pN0-stage LUAD samples (**Figure 3E**).

Furthermore, as an internal validation, we randomly selected (from a prospectively established lung tumor biobank by a pathologist) pN0-stage LUAD patients who survived less (N = 5) or more (N = 5) than 5 years, demonstrating that THBS2 protein expression was significantly higher in pN0-stage I LUAD patients with shorter OS than in those with longer OS (p=0.0024) (**Figure 3F**). Finally, in an independent, prospectively-established, internal LUAD cohort (tissue microarray data; N = 93), the THBS2 protein level was identified as an independent influencing factor of OS (**Figure S5A, B; Table S3**). Notably, in line with the public datasets (**Figure 3; Figure S4**), the THBS2 protein level is significantly higher in the tumors, compared to the matched normal lung tissue (**Figure S5A; Table S3**).

Additionally, we retrospectively examined LUAD samples with pathological confirmation of regional lymph node metastasis, which is closely associated with (regional or distant) tumor recurrence and metastasis. Strikingly, we observed that 6 out of 10 tumor-draining lymph nodes samples had proportions, albeit heterogeneous, of metastatic LUAD cancer cells that were THBS2 positive (**Figure. 3G**), in agreement with our findings showing the association of high *THBS2* expression with tumor relapse (**Figure 2; Figure S3**). Notably, the lymph node represents a critical meeting point of immune cells where adaptive immunity is induced. These lines of evidence support the idea that THBS2 might facilitate tumor recurrence and metastasis, in part, by promoting the escape of immune surveillance in LUAD.

Taken together, the above data reproducibly demonstrated THBS2 as a robust biomarker in predicting the poor prognosis of patients with early pN0-stage LUAD, and high THBS2 expression marks an aggressive phenotype of LUAD.

### THBS2 is highly secreted via exosomes by aggressive LUAD tumors

The above evidence revealed THBS2 as a secreted protein (**Figure S3B**) and also a predictive marker for patients' prognosis and treatment response (**Figure 2; Figure 3; Figure S3-S5**), which was reminiscent of exosomes that are important mediators of cell-to-cell communication and are associated with drug resistance, as well as tumor recurrence/metastasis 14.

**Figure 4:**
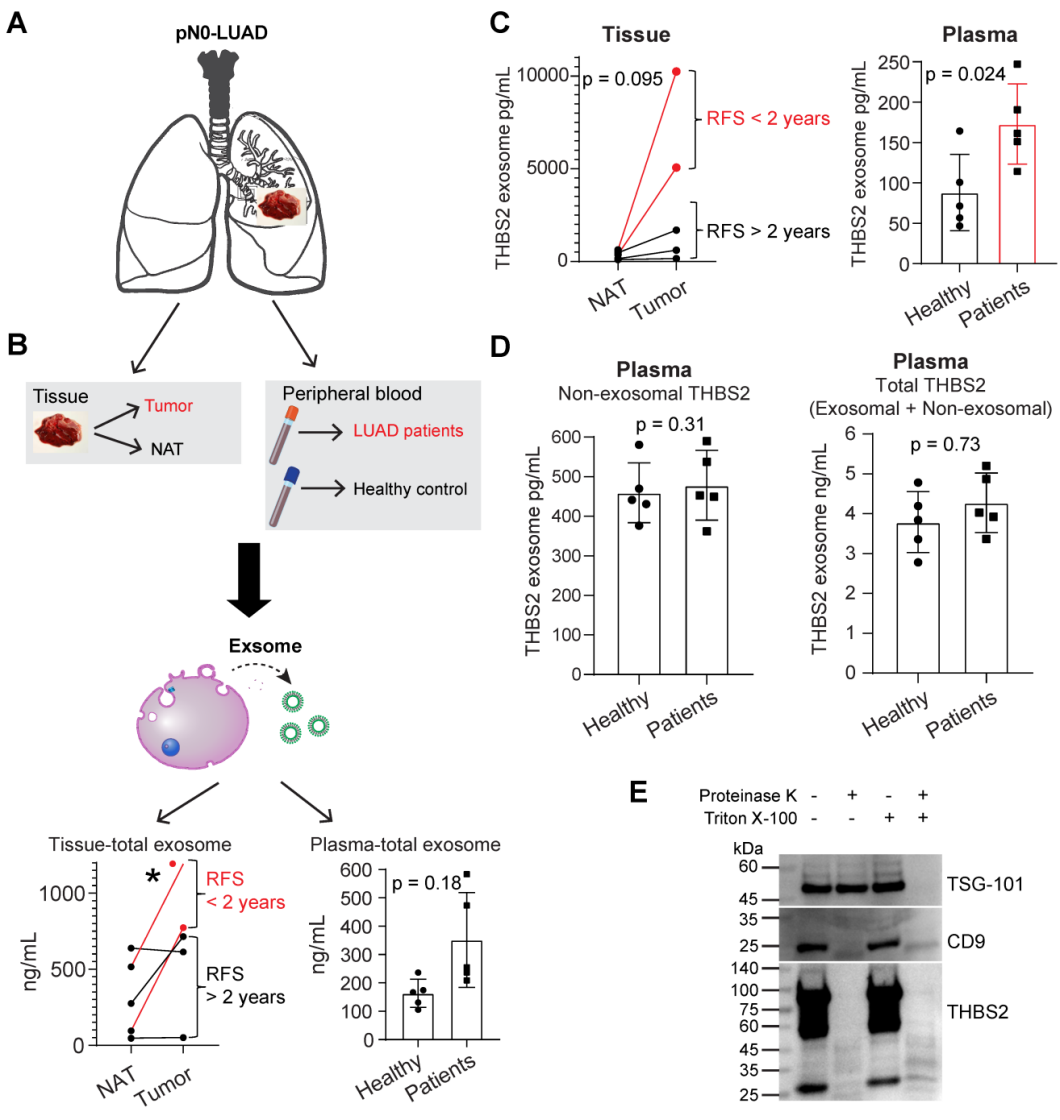
** Identifying secreted THBS2 as an exosome protein.** A, Exosome isolation, purification and characterization. In A, lung tissue (primary lung adenocarcinoma (LUAD, N = 5); normal adjacent lung (NAT, N = 5)) and blood plasma samples (from LUAD, N = 5; from healthy controls, N = 5) were included. B-D, The quantitation of purified total (B) and THBS2 (C) exosome proteins of lung tissue and plasma samples; P-value (*p < 0.05) was calculated using two-sided paired (left panel) or unpaired (right panel) t-test. (D), The difference in the amount of non-exosomal (left panel) and total (exosomal plus non-exosomal; right panel) THBS2 protein between plasma from LUAD and healthy controls (N = 5 for each group). P-value was calculated using two-sided unpaired t-test. E, Dissecting the specific location of exosomal THBS2 using proteinase K assay. 20 mL plasma of 6 LUAD patients were used to purify exosomes (500 μl; concentration: 1062 ng/μL), and were then incubated in PBS (control), proteinase K alone, Triton X-100, or combined proteinase K plus Triton X-100, respectively. These samples were then subjected to immunoblot. TSG-101, a typical exosomal intra-membrane protein, and CD9, a classical exosomal trans-membrane protein, were used as positive controls. Notably, THBS2 was detected only in the membrane but not inside of the exosomes.

We thus purified exosomes from the tumor tissues and plasma samples of pN0-stage LUAD (N = 5) and healthy controls (N = 5), respectively (**Figure 4A, B; Figure S6A, B**), and then performed the quantifications of THBS2 exosomes (**Figure 4C, D**). The data in **Figure S6A, B** validated the purity of isolated exosomes, based on characterizations of the morphology (transmission electron microscopy), and the size measurement (Nanoparticle Tracking Analysis), and classical exosomal markers (western blot). Our data confirmed the abundance of tissue-derived total (**Figure 4B**) and THBS2 (**Figure 4C**) tumor exosomes in lung tumors, compared to the matched normal lung tissue. Particularly, its level was much higher in tumors from patients with short RFS (**Figure 4B, C**). In parallel, we also detected a higher level of THBS2 exosomes in the plasma of patients with pN0-stage LUAD, compared to that of the healthy controls (**Figure 4C**). By contrast, there is no difference in the non-exosomal THBS2 or the total THBS2 in the plasma between pN0-stage LUADs and the healthy controls (**Figure 4D**), highlighting the promises of the exosomal form of THBS2 in defining an aggressive subset of early-stage LUAD. Besides, we also dissected the specific location of exosomal THBS2, which is closely related to the way it interacts with its target cells (see the discussion). The data demonstrated that THBS2 is located on the membrane rather than inside of LUAD-derived exosomes (**Figure 4E**).

### scRNA-seq analysis reveals subsets of CAFs as a cellular source of THBS2 expression in pN0-stage LUAD

**Figure 5:**
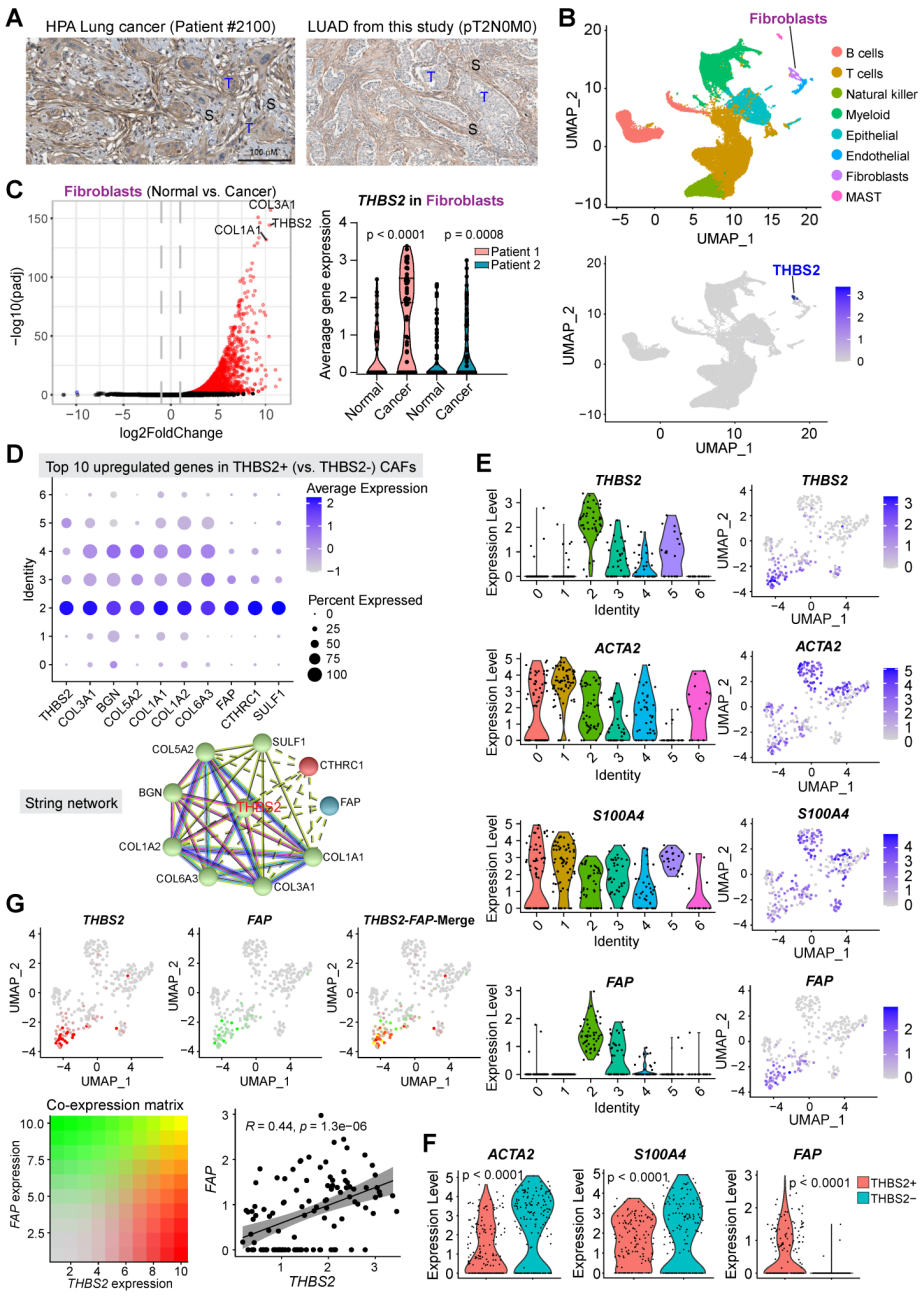
** scRNA-seq analysis reveals subsets of CAFs as the major source of THBS2 production.** A, Immunohistochemistry/hematoxylin staining showing the expression of THBS2 in LUAD samples from TCGA and this study. Of note, THBS2 was detected in cancer and more predominantly in peritumoral stromal cells. Scale bar: 100 μm. B, UMAP (Uniform Manifold Approximation and Projection) plot showing the expression of *THBS2* across different cellular subpopulations from lung tumors (pT1N0M0, N = 2) and matched normal lung tissue (N = 2). Of note, high *THBS2* expression was from fibroblasts based on annotation (see Figure S7B). C, Volcano plot (left panel) showing the differentially expressed genes between fibroblasts from lung primary tumors and normal adjacent lung tissue. Of note, THBS2 was listed as the top genes upregulated in from fibroblasts from lung primary tumors, compared with those from matched normal lung tissue. In the right panel, the violin plots showing that *THBS2* was upregulated in lung primary tumors compared to normal adjacent lung tissue across two individuals. The significance was determined using two-sided Student's t-test. D, The upper panel showing the top 10 genes of THBS2+ compared with THBS2- CAFs across the 7 CAF subclusters. The lower panel showing the String interaction network of the top 10 genes. In the String network, the interactions were clustered (N = 3; represented by 3 colors) based on kmeans clustering algorithm. E, Violin (left) and UMAP (right) plots showing *THBS2, ACTA2, S100A4, or FAP* expression across different CAF subclusters. Of note, THBS2 was mainly expressed by cluster 2-, 5-, 3 and 4-annotated CAFs. F, Violin and UMAP plots showing the difference in the expression of *ACTA2, S100A4, or FAP between THBS2+ and THBS2- CAFs.* P-value calculated by two-sided unpaired t-test. G, UMAP plots showing the co-expression of *THBS2* and *FAP* across individual single CAFs. Red/green dots represent CAFs expressing THBS2+ only/FAP+ only (upper left/middle panels), respectively, and yellow dots indicate CAFs co-expressing THBS2 and FAP only (upper right panel); lower left panel showing the co-expression matrix across CAFs with different expression of FAP and THBS2; lower right panels showing the correlation (Pearson) between THBS2 and FAP across THBS2+ CAFs. (1) Strong correlation: Pearson`s r ≥ 0.8; (2) Moderate: 0.5 ≤ Pearson`s r < 0.8; (3) Weak: 0.3 ≤ Pearson`s r < 0.5; (4) No correlation: Pearson`s r < 0.3). The darker the color, the higher the expression level of the indicated markers. Of note, only a minority of CAFs co-express high THBS2 and FAP.

Next, we sought to identify the cellular source of THBS2 expression. Immunohistochemistry (IHC) analysis demonstrated that THBS2 was detectable in cancer cells but prominently in peritumoral stromal cells (**Figure S3A**; **Figure 5A**). In our WGCNA analysis, we noted that THBS2 was mostly co-clustered with fibroblast activation protein (FAP) (**Figure 2E**), a typical marker of fibroblasts, prompting us to hypothesize that CAFs might be a major source of THBS2 production.

Previous evidence highlighted the heterogeneity CAFs, defining distinct CAF subsets characterized by different molecular profiles, biological functions, and tumor immunological signatures 73, 74. To investigate whether CAFs are the major source of THBS2 expression and to identify the specific subsets of CAFs, we applied the single-cell RNA sequencing (scRNA-seq) analysis, a powerful tool deconvolving the cell-type composition within tissues and deciphering the transcriptomic profiles of each cell, to two surgical cases with early-stage lung cancer (pT1N0M0) and adjacent normal lung tissues (**Figure S7A, B**). Notably, in these samples, scRNA-seq data showed a small proportion of CAFs but a large proportion of immune cells (**Figure 5B**). These observations were partially due to the bias introduced during tissue dissociations, leading to overestimating the immune cell proportions in comparison to the stromal and epithelial cell types 75.

The results demonstrated that THBS2 was mainly expressed by fibroblasts (**Figure 5B; Figure S7B**), and more importantly, fibroblasts from tumors (CAFs) had significantly higher expression of *THBS2* than matched normal lung-derived fibroblasts (**Figure 5C**). Given the heterogeneity of CAFs 73, 74, we further divided CAFs into 7 subclusters, based on the Clustree algorithm that is used to increase gene representation and achieve optimal cluster separation (**Figure S7C**) 27, we found that in the two studied lung tumor samples, THBS2 was mainly expressed by four CAF subclusters (2, 3, 4, 5, particularly 2) (**Figure S7D, E**), suggesting the presence of heterogeneity within THBS2+ CAFs. Furthermore, the top 10 upregulated genes in THBS2+ CAFs, compared with THBS2- CAF subclusters, were (from top 1 to top 10): * THBS2, COL3A1, BGN, COL5A2, COL1A1, COL6A3, FAP, CTHRC1* and *SULF1* (**Figure 5D**).

CAFs are comprised of highly heterogeneous populations 73, 74, and can be generally defined using a panel of typical markers, e.g. FAP, αSMA+ (myofibroblast-like; encoded by *ACTA2*), etc. We then sought to know whether THBS2+ CAFs represent a unique population that is different from CAFs defined by classical markers. Strikingly, based on the scRNA-seq data of CAFs, we observed that THBS2+ CAFs appear to have a drastically different distribution from ACTA2+ or S100A4+ CAFs (**Figure 5E**), and THBS2+ CAFs have significantly lower expression of *ACTA2* or *S100A4* than THBS2- CAFs (**Figure 5F**). Furthermore, co-expression analyses demonstrated that FAP-high CAFs are largely not overlapping with THBS2-high CAFs, and that there is a significant but a weak correlation between FAP and THBS2 across individual CAFs (**Figure 5G**), although overall THBS2+ CAFs have higher expression of FAP than THBS2- CAFs (**Figure 5F**).

Additionally, we mined public datasets relating to scRNA-seq analysis of non-small cell lung cancer (NSCLC) samples from TISCH, a comprehensive web resource enabling interactive single-cell transcriptome visualization of the TME 76. The analyses also revealed that *THBS2* was consistently expressed by CAFs **(Figure S7F)**. Moreover, these observations also held true in other organs-derived tumors (e.g., breast invasive carcinoma [BRCA], Head and Neck squamous cell carcinoma [HNSCC], ovarian serous cystadenocarcinoma [OV], pancreatic adenocarcinoma [PAAD], Bladder Urothelial Carcinoma [BLCA] sarcoma [SARC]) that are generally characterized by a high ECM/stromal signature and poor response to immunotherapy (**Figure S8**) 77. The above evidence suggested that THBS2 expression is mainly derived from CAFs, which is independent of organ lineage.

Collectively, these data indicated that THBS2+ CAFs represent a unique subpopulation that did not co-cluster with CAFs defined by typical markers.

### THBS2-high LUAD is characterized by a high ECM/stromal signature together with suppressive tumor immunity

**Figure 6:**
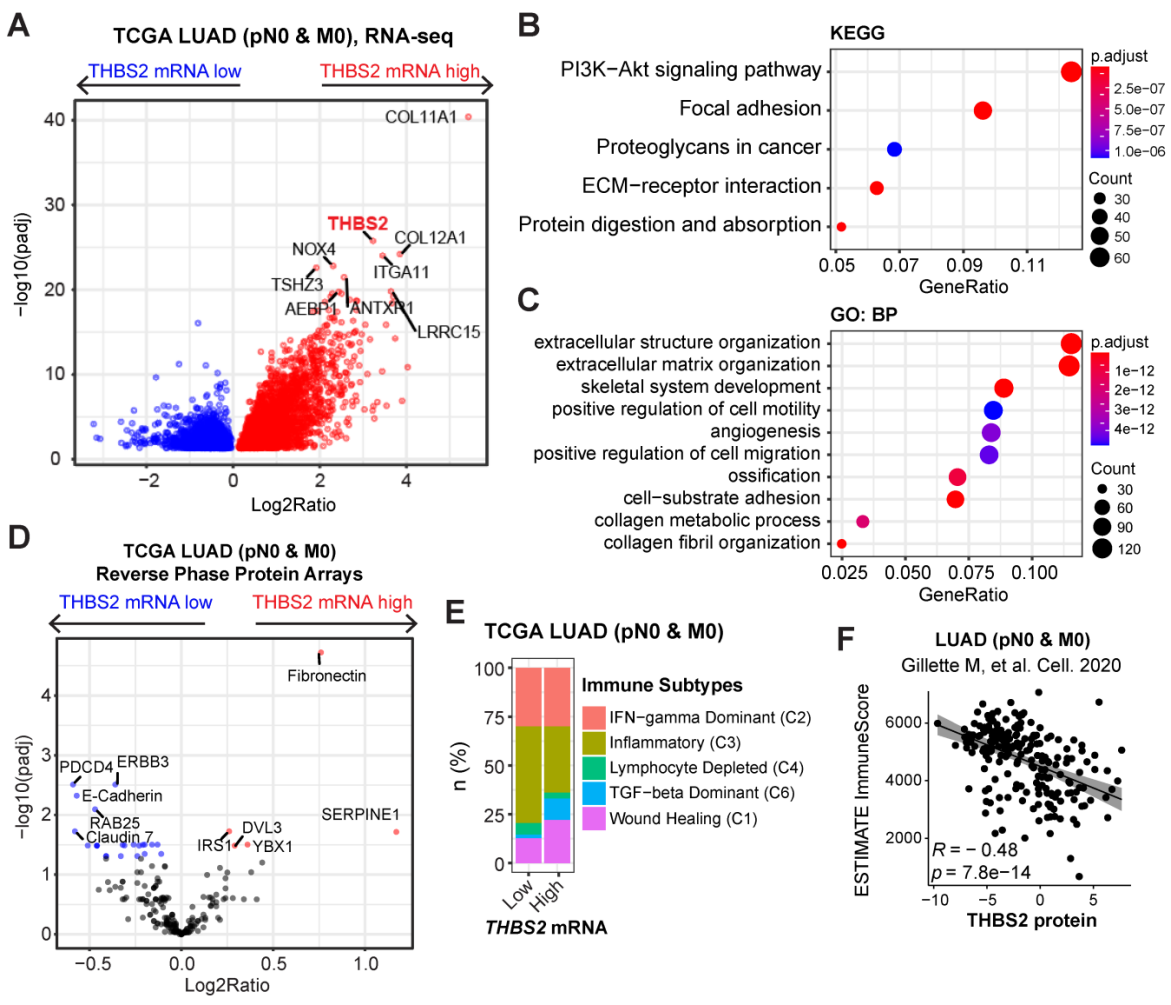
** LUAD tumors with high THBS2 expression are characterized by an enriched ECM/stromal signature and dysregulated tumor immunity.** A-C, Pathway enrichment in THBS2-high LUAD compared with THBS2-low LUAD. A, Volcano plot showing the upregulated genes in THBS2-high LUAD. Data were from TCGA pN0-stage LUAD. B, C, KEGG (Kyoto Encyclopedia of Genes and Genomes; B), and GO-BP (Gene Ontology Biological Processes; C) pathway analyses were performed based on A. D, Differentially expressed proteins between THBS2 (mRNA)-high and THBS2-low LUAD based on A. The blue color indicates the significantly downregulated proteins (adjusted p < 0.01), while the red color represents the significantly upregulated proteins in THBS2-high LUAD. Data were downloaded and reanalyzed from the TCGA LUAD RPPA (reversed-phase protein array) dataset (see the details in Methods). E, Difference in the distribution of immune subtypes (C1-C6) between THBS2-high and THBS2-low LUAD based on A. The genes contained in each signature were evaluated using model-based clustering by p the “mclust” R package. Each sample was finally grouped based on its predominance with the C1-C6 signature. The immune subtype models were based on Thorsson V et al. Immunity. 2018 (See the methods). F, The correlation between the antitumor immune score and THBS2 protein level across pN0-stage LUAD. Data of antitumor immune score and THBS2 protein level were downloaded and reanalyzed from the Supplementary Tables published in Gillettle M, et al. Cell. 2020.

To investigate the biological functions of THBS2 in LUAD, we analyzed the molecular features of THBS2-high LUAD samples. Based on the RNA-sequencing data of TCGA pN0-stage LUAD, we observed that THBS2-high LUAD tumors were characterized by enrichment for PI3K-AKT, focal adhesion, proteoglycan ECM-receptor interaction, and protein/collagen metabolic pathways/process, positive regulation of cell motility, and stromal signature (**Figure 6A-C**). Similarly, reverse phase protein array (RPPA) data (including 216 tumorigenesis-associated proteins) of TCGA pN0-stage LUAD showed that THBS2-high LUAD tumors are mostly characterized by a high level of fibronectin, a typical mesenchymal marker, and low level of several classical epithelial biomarkers, E-cadherin, ERBB3 and Claudin-7 (**Figure 6D**). These analyses THBS2-high LUAD is characterized by a high ECM/stromal signature that marks a mesenchymal-like phenotype and is typically associated with tumor metastasis and therapy resistance.

In comparison with the scRNA-seq profilings of THBS2- CAFs, gene set enrichment analysis (GSEA; Hallmark module) revealed that THBS2+ CAFs were significantly enriched for signatures of the epithelial-to-mesenchymal transition (EMT), immune-inflammatory response (e.g., TNFα-NFκB, IL6-JAK-STAT3, IL2-STAT5, inflammatory response; complement, interferon-gamma response, allograft rejection), hypoxia, TGF-β and glycolysis signaling pathways (**Figure S9**), suggesting a pleiotropic role and an immune-inflammatory phenotype of THBS2+ CAFs.

Considerable evidence has revealed that tumors with a high stromal signature are prone to immune evasion and resistance to immunotherapy and that tumor-derived glycoproteins endow immunosuppressive functions 78. Intriguingly, based on curated immune subtype models 58, our analysis showed that pN0-stage LUAD tumors with high *THBS2* gene expression were enriched for TGF-beta Dominant and Wound Healing immune subtypes but reduced for Inflammatory subtypes (**Figure 6E**). In support of this, based on the proteomics data of pN0-stage LUAD tumors 50, THBS2 protein level was significantly negatively correlated with the antitumor immune score (**Figure 6F**). These lines of evidence reinforced the notion that THBS2-enriched TME might facilitate immune escape (**Figure 3G**).

### Multiplexed IHC (mIHC) staining analysis revealed the spatial expression of THBS2 and its interaction with TIME

**Figure 7:**
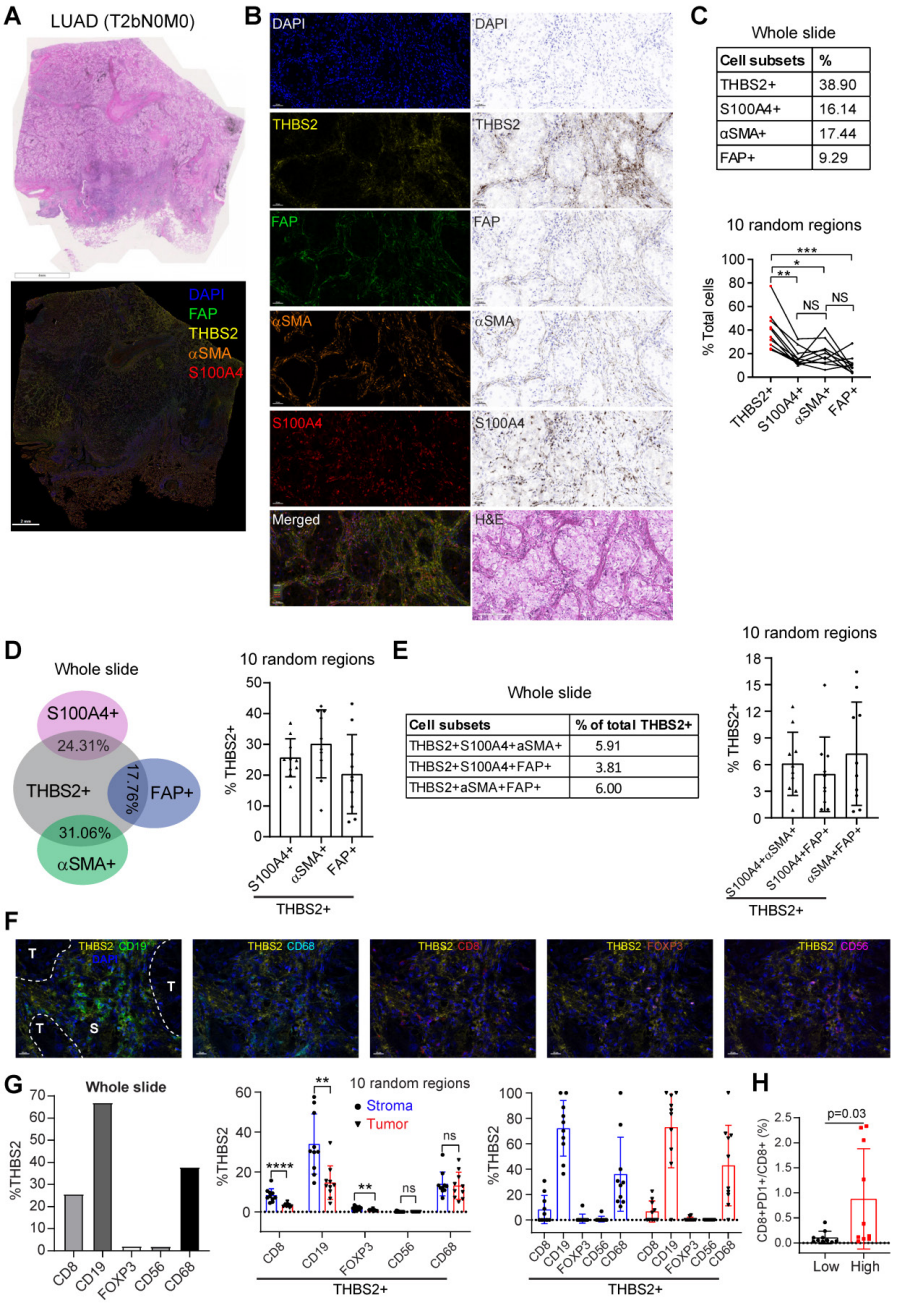
** Multiplexed IHC shows the spatial association between THBS2 and typical CAF markers in LUAD.** A, B, THBS2 co-staining with three classical CAF markers (αSMA, FAP, S100A4 [FSP1]) in a resected LUAD (lung adenocarcinoma) (pT2bN0M0) sample. The image acquisition of all markers occurred simultaneously. Panel A shows the whole slide scan; panel B shows the representative regions (200x). Scale bar: 50 μm. C-E, Individual or selected combinations of THBS2 and three classical CAF markers (αSMA, FAP, S100A4 [FSP1]) markers (whole slide and 10 randomly selected regions) were quantified and shown. Single-staining (THBS2, αSMA, FAP, or S100A4; **C**), double-staining (THBS2/αSMA, THBS2/FAP, or THBS2/S100A4; **D**), triple-staining (THBS2/αSMA/ FAP, THBS2/FAP/S100A4, or THBS2/αSMA/S100A4; **E**) were quantified by using HALO® software (Please see the detailed description in the Methods section “Image acquisition and data quantification”). *p < 0.05; **p < 0.01; ****p < 0.0001 by paired ANOVA test. F, G, Indicated combinations markers (whole slide [left] and 10 randomly selected [under 50x magnification] regions [middle and right]) were quantified and shown. The upper panel (F) showed the representative regions (400x). In tumor and stromal compartments within the 10 different regions, the expression of indicated markers was quantified and compared, respectively. **p < 0.01; ****p < 0.0001 by two-sided student`s t-test. Scale bar: 20 μm. H, Ratio of CD8+PD1+ to total CD8+ T cells in 10 randomly (5 tumoral and 5 stromal regions) selected regions of a LUAD tumor from A (related to Figure S15C). We performed multiplexed IHC with CD4, CD8, CD19 and PD-1 (5-Color Multiple IHC Kit) from a serial slide of the same tumor as Figure 7A-G. p-value was calculated using paired student`s t-test.

Next, we investigated the interaction between THBS2 and tumor immune microenvironment (TIME) by implementing mIHC staining analysis that enables simultaneous multiparametric readouts at the single-cell level from a single tissue section. First, to confirm the spatial expression of THBS2 (**Figure 5E-G**), we performed mIHC analysis by co-staining THBS2 and three typical CAF markers (FAP, αSMA and S100A4 [also known as FSP-1]) (**Figure 7A**), the results confirmed that THBS2 and the three classical CAF markers were mainly expressed in peritumoral stromal compartments (**Figure 7B**). Overall, in the tested LUAD (pT2bN0M0) samples, the percentage of THBS2+ CAFs was much higher than that of FAP+, αSMA+ or S100A4+ CAFs in the peritumoral stromal compartments (**Figure 7B, C**). Notably, only a minority of THBS2 overlapped with the three classical CAF markers (**Figure 7D-E**), supporting that THBS2+ CAFs represent a unique subset, which was in line with the above scRNA-seq data (**Figure 5E-G**).

In parallel, our findings were examined in a resected sample with lung squamous cell cancer (LUSC, pT2bN0M0) that has the same histological grade (moderate) and similar genetic backgrounds (no common oncogenic mutations in NSCLC) (**Figure S10A-E**), given that there is a dramatic difference in the TME between LUAD and LUSC 79. In contrast to LUAD, the percentage of THBS2+ CAFs in the LUSC sample was much lower than that of FAP+, αSMA+ or S100A4+ CAFs in peritumoral stromal compartments (**Figure S10C**). Similar to LUAD, only a few THBS2 overlapped with the three classic CAF markers in the LUSC sample (**Figure S10D, E**). Based on the TCGA NSCLC cohort, pN0-stage LUSC tumors have significantly higher THBS2 than the LUAD counterparts (**Figure S10F**). Concerning patient prognosis, high *THBS2* expression also predicted poor survival in pN0-stage LUSC after surgery (**Figure S11**). Whether the biological functions of THBS2 in LUAD differ from those in LUSC warrants further study.

Our above evidence revealed a potential link of THBS2 with TIME. CD36 and CD47 are two well-characterized receptors of secreted THBS2 80. Interestingly, recent studies demonstrated that CD36 was selectively upregulated intratumoral regulatory T (Treg) cells 81 and promoted intratumoral CD8+ T cell dysfunction 82. Thus, targeting CD36 could enhance the response to immunotherapy 81, 82. Similarly, CD47, which promotes immune evasion by engaging signal-regulatory protein alpha (SIRPα) and serves as an inhibitory receptor on macrophages, is emerging as a novel macrophage immune checkpoint for cancer immunotherapy 83. Our scRNA-seq profiling together with the mining data showed that CD36 was mainly expressed by monocytes/macrophages while CD47 was broadly expressed particularly by cancer cells **(Figure S12A, B)**. More intriguingly, inferring cell-cell communication using the CellChat algorithm revealed THBS2+ CAFs have more profound interaction weights with both tumor and immune cells compared with THBS2- CAFs based on our scRNA-seq of two pT1N0M0 lung cancer samples (**Figure S13A**). Further, the ligand-receptor analysis showed THBS2-CD47 was likely to contribute mostly to the ligand-receptor-based interactions (**Figure S13B**). Among the top enriched signaling pathway network (**Figure S13C**), the interactions between THBS2+ CAFs and other cells within the tumor micro-ecosystem were most mediated by THBS, followed by stromal/immune signaling pathways, e.g. THY-1, FN-1, TGF-β, IL6/4, MHC-I, VEGF, CD40, or TIGIT. These data suggest a pleiotropic role of THBS2 in the TME of early-stage LUAD. Since our above evidence demonstrated that THBS2 could be shuttled in the form of non-exosome and exosomes, thus supporting that THBS2 can interact with immune cells via ligand-receptor recognition or vesicle uptake, consequently affecting their respective functions.

**Figure 8:**
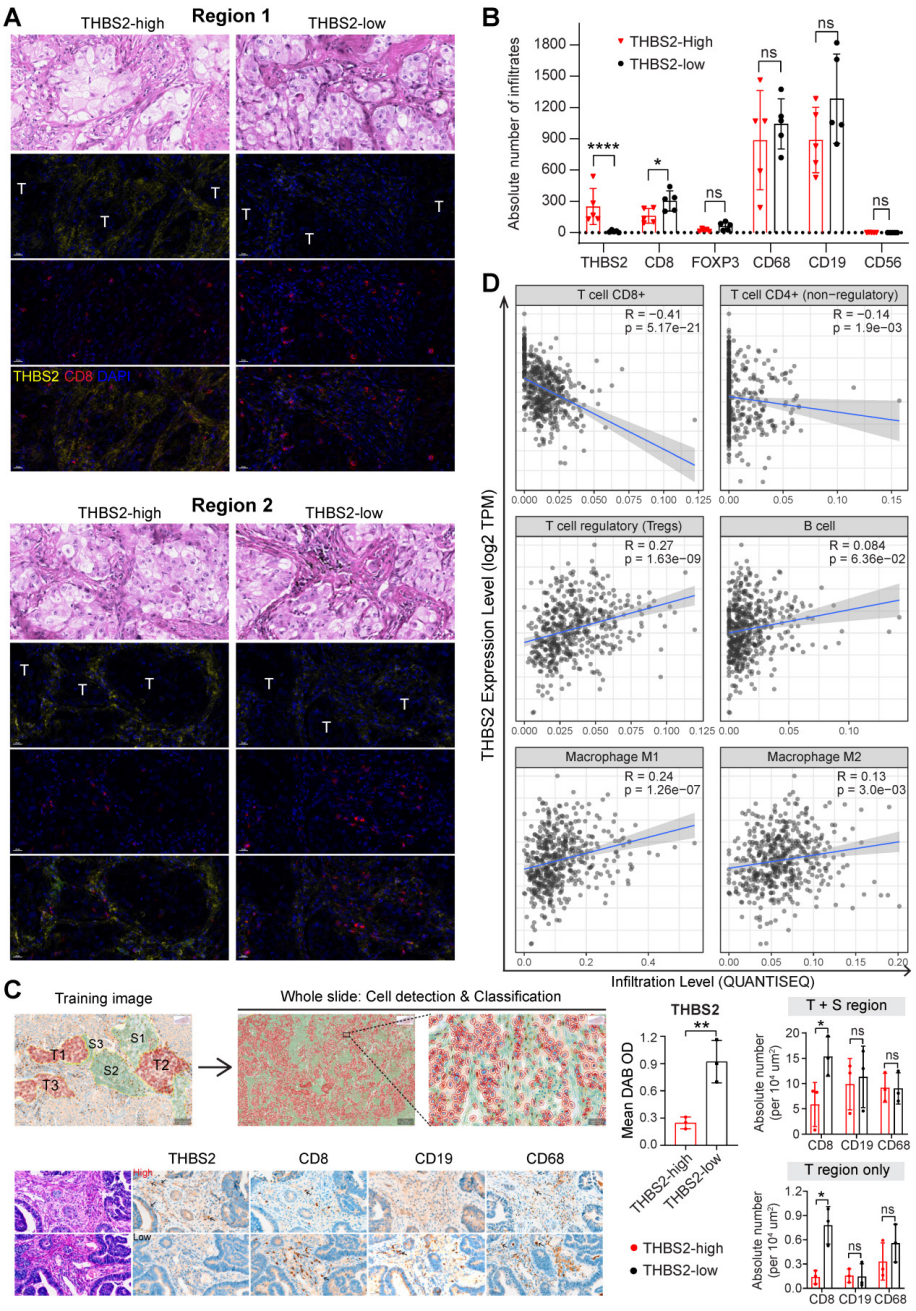
** The association between THBS2 expression and immune infiltrates in LUAD.** A, B, Panel A showing the representative images of THBS2 co-staining with CD8 T lymphocytes in two regions (THBS2-high and -low) of a resected LUAD (lung adenocarcinoma) (pT2bN0M0) sample. The image acquisition of all markers occurred simultaneously with the representative regions were shown (200x). Panel B showing the quantification (absolute number of the indicated positive cells) of 5 random regions (under 50x). The quantification of THBS2 level in the first barplot was shown to confirm the difference in the expression of THBS2 between THBS2-high and -low groups *p < 0.05; ****p < 0.0001 by two-sided student`s t-test. ns, not significant. Scale bar: 20 μm. C, An independent pN0-stage LUAD cohort (N = 6) was used to compare the difference in immune cell infiltrates between THBS2-high (N = 3) and -low (N = 3) groups. Left upper panels showing the tumor (T, in red) and stromal (S, in blue) cells (100X), which were used for training to recognize the differential features between tumor and stromal cells (using QuPath software, version 0.3.2). Then the established unique parameters of tumor and stromal cells were applied to the entire slide (left lower). Left lower panels showing the representative IHC images (200x, left). The right panels showing the quantifications of the indicated protein markers. *p < 0.05; **p < 0.01 by two-sided student`s t-test. ns, not significant. Scale bar: 50 μm. D, Correlation between THBS2 expression and immune infiltrates across p-N0 stage LUAD from TCGA cohort.

We further co-stained THBS2 with markers of cytotoxic T (CD8), Treg (FoxP3) and B (CD19) lymphocytes, as well as tumor-associated macrophages (CD68) 84 and nature killer (NK) cells (CD56) 85, which showed that immune cells were mainly located at the stromal compartment (**Figure 7F; Figure S14A**). Strikingly, among the biomarkers examined, CD19, followed by CD68 and CD8, were the most co-stained markers with THBS2 (**Figure 7F, G**), suggesting that THBS2 mainly impacts B cells, macrophages and CD8+ T cells within TIME, thus potentially modulating tumor immunity via interacting with these immune cells. Additionally, THBS2-expression high compartments were associated with elevated percentage of CD8+PD1+ (p=0.03), CD4+PD1+ (p=0.07), and CD19+PD1+ (p=0.14) lymphocytes (**Figure 7H; Figure S14B; Figure S15D**), indicating that THBS2 might promote exhaustion of these infiltrated immune cells. Furthermore, quantification of immune cell infiltrates revealed a significant decrease in CD8+ T cells (**Figure 8A-C; Figure S15A, B**), to a less extent in CD19+ B cells accompanied by high THBS2 expression (**Figure 8B, C; Figure S15B, C**). In agreement, estimation of the immune cell infiltrates, using quanTIseq, an algorithm specifically developed for RNA-sequencing data 60, showed a significantly negative correlation between CD8+ T cell infiltrates and THBS2 expression across TCGA LUAD samples (**Figure 8D**). There is also a correlation, albeit to a less extent, between THBS2 expression with other immune cell infiltrates (**Figure 8D**). Together, these data support a suppressive role of THBS2 in modulating TIME; however, how THBS2 exactly affects these immune cells remains to be further elucidated.

### THBS2-high LUAD displays a poor response to clinical immunotherapy

The above evidence revealed a suppressive TIME in THBS2-high LUAD, prompting us to investigate the association between THBS2 expression and the response to clinical immunotherapy.

**Figure 9:**
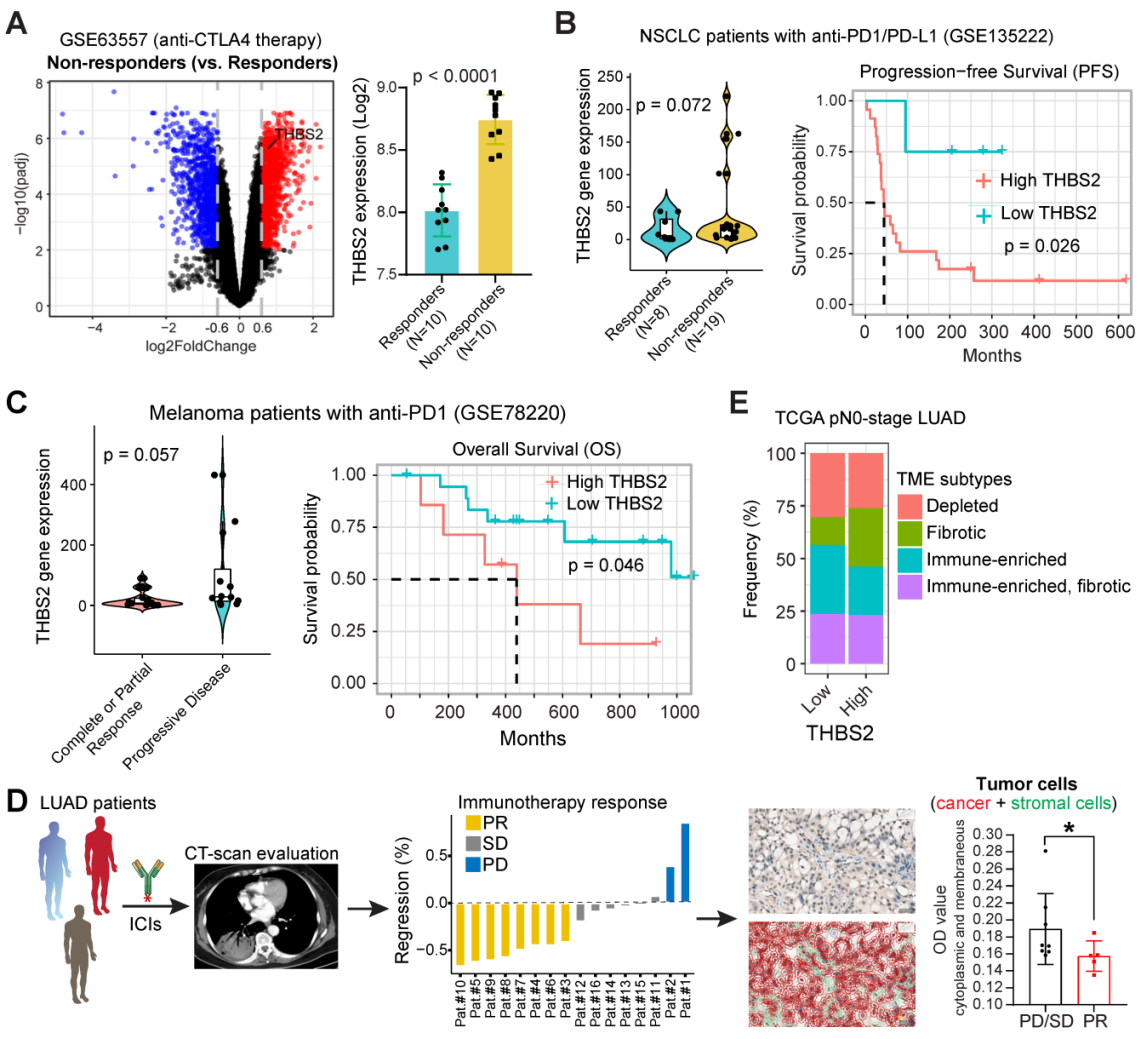
** THBS2 expression effectively predicts the response to immunotherapy in clinical patients.** A, Differentially expressed genes between the tumors that respond and do not respond to anti-CTLA4 immunotherapy. A barplot was shown to the right specifically illustrating the difference between the responders and non-responders. Of note, THBS2 was significantly highly expressed in tumors that did not respond to anti-CTLA4 immunotherapy. Data were downloaded and reanalyzed from the GSE63557 dataset. B, C, External cohorts validating the association between THBS2 expression in pretreated tumor samples and anti-PD1/PD-L1 therapy from non-small cell lung cancer (NSCLC) (GSE135222; B) and melanoma (GSE78220; C) cohorts. The left panel showed the difference in THBS2 expression between the responders and non-responders; the right panel shows the association of THBS2 in pretreated samples with PFS of patients in this cohort. In the melanoma cohort, the data of patient 16 were excluded because of the on-treatment biopsy. Of note, in the left panel of B, the dashed line was used to highlight that in this studied cohort, the tumors whose baseline expression of THBS2 > 50 all belong to the non-responders group. However, in the right panel, the THBS2 expression in the survival plot was grouped based on the optimal cutoff value determined by R software (see the Methods), but not the dashed line. Likewise, a similar group strategy was used in the two panels of Figure 9C. D, The workflow showing the evaluation of responses to immune checkpoint inhibitors (ICIs) in LUAD patients. After at least two cycles of ICIs, the therapeutic response was evaluated by using computed tomography (CT) scans based on the guideline of RECIST 1.1 (see the Methods). Immunohistochemistry-based quantifications (using Qupath software, see the methods) showing the association between THBS2 expression in pretreated LUAD biopsies and therapeutic response to anti-PD1 immunotherapy. PR: partial response; SD: stable disease; PD: progressed disease. Of note, we acquired a total of 16 patients` samples (middle panel), of which, 3 samples from the PR group could not be evaluated due to the small size and poor quality of the biopsies. As a result, we could not collect the quantification data from these 3 samples, and only 13 samples were finally included for analysis. Scale bar: 20 μm. E, Difference in the distribution of the tumor microenvironment signature (from Bagaev A, et al. Cancer Cell. 2021) between THBS2-high and THBS2-low tumors based on pN0M0-stage TCGA LUAD.

First, we performed integrative analyses of the transcriptomic and therapeutic response data of 20 BALB/c mice inoculated subcutaneously with AB1-HA cells and treated with anti-CTLA4 therapy (GSE63557) 57, and intriguingly, tumors from non-responders had significantly upregulated THBS2 expression (**Figure 9A**). Second, along the same line, mining two independent datasets that contain lung cancer (GSE135222) or melanoma (GSE78220) patients treated with anti-PD1/PD-L1 immunotherapies, we observed that higher THBS2 expression was associated with poor response and short PFS/OS after immunotherapy (**Figure 9B, C**). Additionally, we retrospectively reviewed THBS2 expression in the pre-immunotherapy biopsies of LUAD patients (N = 13) who subsequently received anti-PD-1 immunotherapy after the failure of first-/second-line treatment. Remarkably, low expression of THBS2 was significantly associated with a better response to immunotherapy (**Figure 9D**). Finally, we integrated the recently established four TME subtypes that deciphered the abundance of both malignant and non-malignant cell subpopulations and the activity of tumor-promoting and tumor-suppressive processes occurring within a tumor 59. This new classification model demonstrated robustness in identifying subsets responding to immunotherapy. We applied the four TME subtypes to the above lung cancer patient cohort receiving immunotherapy (GSE135222) and observed that THBS2 expression was significantly positively correlated with Angiogenesis-Fibrosis subtype (mainly derived from the CAF signature) and EMT signature (**Figure S16**). In contrast, THBS2 expression was significantly negatively correlated with the antitumor immune microenvironment (specifically effector T cells and NK cells) (**Figure S16**). Consistently, in TCGA pN0-stage LUAD, high THBS2 expression was associated with more Fibrotic but less Immune-enriched TME subtypes (**Figure 9E**).

Together, these lines of evidence suggested that LUAD tumors with high expression of THBS2 are prone to immune escape and resistance to immunotherapies.

### THBS2 suppresses *ex vivo* T cell proliferation and promotes tumor growth and metastasis in LUAD xenografts

**Figure 10:**
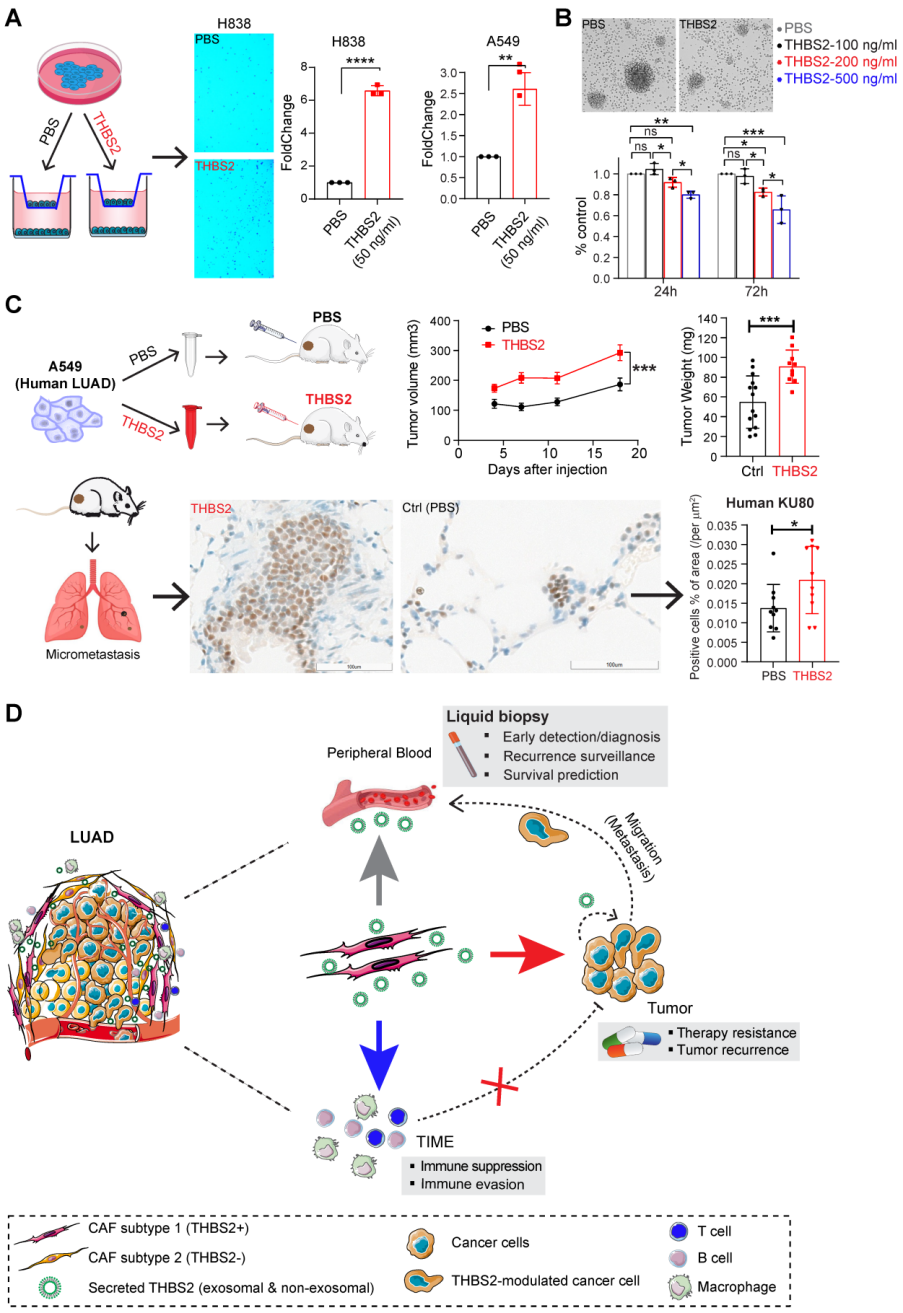
** THBS2 plays a pleiotropic role in modulating cancer cells and immune cells.** A, *In vitro* Transwell assays showing the effect of THBS2 on the migration of human A549 and H838 LUAD (lung adenocarcinoma) cells. Representative images (10x) of H838-Transwell assays were shown in the middle. ****p < 0.0001 by two-sided student`s t-test. B, *Ex vivo* T cell proliferation assay showing the effect of THBS2 (96h) on the proliferation of activated CD3+ T cells. The representative image on the top is under the treatment 500 ng/mL THBS2 recombinant protein. C, *In vivo* LUAD xenografts showing the effect of THBS2 on subcutaneous tumor growth (upper panel) and micro-metastasis to the lung (lower panel), with the quantifications shown to the right. Human-specific KU80 antibody was used to detect human-derived A549 LUAD cells. To make the analysis comparable, the number of positive cells was normalized to the scanned area (per μm^2^). ***p < 0.001 by two-way ANOVA test (tumor volume). **p < 0.01; ***p < 0.001 by two-sided student`s t-test. D, A hypothetical model illustrating the pleiotropic roles of THBS2 in the micro-ecosystem of LUAD. 1) Secreted THBS2 can be detected in peripheral blood, thus as a promising liquid biomarker; 2) THBS2 promotes tumor recurrence/metastasis/treatment resistance; 3) THBS2 promotes an immune-suppressive microenvironment by interacting with immune cells, thereby facilitating the immune escape of LUAD tumor cells. Figures were created with BioRender.com. TIME: tumor immune microenvironment. CAF: cancer-associated fibroblasts.

Because THBS2 is a protein secreted by CAFs into the tumor microenvironment and correlates with tumor recurrence, we treated cancer cells with exogenous recombinant THBS2 protein to mimic the effect of THBS2 on LUAD cancer cells and T cells isolated from a LUAD patient. *In vitro* transwell migration assays showed that the presence of THBS2 (50 ng/mL, the average level in the serum of patients with early-stage lung cancer 64) dramatically facilitated the migration of LUAD cells (**Figure 10A; Figure S17A**), although THBS2 does not promote their proliferation (**Figure S17B**). Besides, co-incubation with THBS2 for 96h (200-500 ng/mL, lower than the maximum level secreted by aggressive LUAD tissue (**Figure 4C**)) suppressed the *ex vivo* proliferation of isolated and activated CD3+ T cells from a LUAD patient (**Figure 10B**). In addition, we subcutaneously co-injected THBS2 recombinant protein together with human LUAD A549 cells into immune-deficient nude mice (**Figure 10C**) and mouse LLc cells into immune-competent C57BL/6 mice (**Figure S17C**), which promoted a significant increase in tumor growth and distant micro-metastasis to the lung, compared with the control group (**Figure S17D**). The potentially pleiotropic roles of THBS2 in the micro-ecosystem of LUAD were summarized in **Figure 10D**.

## Discussion

pN0-stage LUAD represents a heterogeneous population. Although the primary tumor is radically resected at an early stage, a subset of patients has a high incidence of tumor relapse, resulting in compromised survival 86; however, little is known about the underlying molecular underpinnings. Furthermore, there is little knowledge about the best strategy, e.g., chemotherapy, radiotherapy, targeted therapy or immunotherapy, to manage this high-risk subset following surgery, and whether additional therapeutic interventions are needed remains controversial 8-11. These dilemmas underscore the need to understand the cellular and molecular mechanisms in primary lesions that are prognostic for recurrence and as biomarkers as well as potential targets for intervention. Here, based on the integrated multi-omics data, our study demonstrated that THBS2, preferentially secreted via exosomes by a specific subset of CAFs within lung tumors, represents a promising molecular biomarker to stratify the high-risk subset of patients with pN0-stage LUAD after curative surgery. Besides, our evidence revealed a major role of THBS2+ CAFs in modulating the TIME, consequently conferring an aggressive phenotype.

THBS2 is a disulfide-linked homotrimeric glycoprotein that mediates cell-to-cell and cell-to-matrix interactions. It has been reported that THBS2 is a prognostic biomarker, either poor or good, in a variety of human cancers, including lung cancer 67-69. THBS2 has been largely described as a serum diagnostic biomarker, particularly in pancreatic cancer, which is characterized by high stromal compartments 66. Furthermore, recent evidence detects THBS2 as a cancer-specific exosome protein 87. Additionally, previous evidence revealed the promise of THBS2 as a candidate diagnostic biomarker for early-stage lung cancer 64. In this study, we particularly showed that THBS2 was preferentially secreted via exosomes by the lung tumors displaying a high aggressive phenotype (**Figure 4; Figure S6**), thereby suggesting exosomal THBS2 as a biomarker defining the high-risk subset of LUAD patients at an early stage. Besides, our data also showed that high expression of THBS2 predicts poor survival in pN0-stage LUAD patients treated with adjuvant chemotherapy or EGFR-TKIs, suggesting that this high-risk subset might not be able to benefit from conventional treatment strategies and that novel treatments are needed. Collectively, the evidence from the literature and our study highlights the promise of THBS2 in the early detection of LUAD, dynamically monitoring tumor recurrence and predicting prognosis, as well as facilitating the decision-making of adjuvant therapy management after curative surgery.

Previous evidence did not dissect which exact cellular subtypes within the tumor micro-ecosystem contribute to the production of THBS2. We provided the first evidence showing that THBS2 was mainly derived from specific subsets of CAFs, informed by the scRNA-seq data, which were different from subsets defined by canonical CAF markers **(Figure 5E-G; Figure S7)**. CAFs are the major player promoting therapy resistance and tumor progression. Recently, mounting evidence has highlighted the high heterogeneity of CAFs 73, 74, which play tumor-promoting roles or tumor-restraining functions, highlighting the need to design subtype-specific therapies. Notably, no single marker can define the full CAF populations. A growing list of markers, e.g., αSMA, S100A4/fibroblast specific protein 1 (FSP‐1), FAP and so on, have been used to define activated CAFs. Thus, different markers defined distinct CAF subsets with their unique gene signatures and functions 73, 74. In this study, our work supports that THBS2+ CAFs are mainly expressed in a few subclusters of CAFs and might represent a unique CAF subset that is different from classical CAFs defined by FAP, FSP-1 and αSMA. Whether THBS2+ CAFs have different biological functions remains to be defined.

We further revealed the potential functions of THBS2+ CAFs, indicating that this subset possesses a potential capacity to modulate the cancer cells and, in particular, TME. Our findings were in line with previous evidence demonstrating that glycoproteins played a role in modulating immunity 78 and that subsets of CAFs are a major source of immunosuppressive activity in the TME 73, 74. Concerning its implications for clinical immunotherapy, we observed a negative association of THBS2 expression with the response to immunotherapy. As such, strategies targeting THBS2+ CAFs might be a potential treatment strategy for this aggressive subset. Recent attempts to therapeutically target CAFs (specifically depleting αSMA+ CAFs) failed in preclinical models of pancreatic cancer and even potentially worsened patients` prognosis 88, which was, in part, due to an incomplete understanding of CAF heterogeneity. Targeting other populations of CAFs instead of αSMA+ in a specific context might provide benefits. Together, we provided the first evidence that targeting THBS2+ CAFs might have promises to improve the patients` prognosis and response to multiple therapeutics.

Beyond defining an aggressive subset of early-stage LUAD, we also observed that a high expression of THBS2 predicts poor prognosis in LUAD patients treated with clinical chemotherapy, EGFR-TKIs-targeted therapy or immunotherapy (**Figure S4B, S4E; Figure 9**), which warrants further validations with large clinical cohorts. Nevertheless, these observations suggest a potential role of THBS2+ CAFs in promoting treatment failure, which is in line with the increasingly essential role of CAFs in therapy resistance 74, 89. Further, it remains to be defined whether THBS2 itself or other proteins/components secreted by THBS2+ CAFs contribute to that phenotype.

Our evidence has shown that THBS2 has been shuttled via exosomes (**Figure 4; Figure S6**) 87, and exosome-mediated intercellular communication predominantly occurs in three ways 90: 1) exosome membrane protein can directly bind to the membrane protein of target cells, leading to activating the signaling pathway; 2) in the extracellular matrix, a protease can cleave the exosome membrane protein, and then bind to receptors on the cell membrane of target cells, consequently activating the downstream signaling pathway; 3) the exosome membrane can directly fuse with the cell membrane of target cells, releasing its contents (e.g., proteins, RNA or DNA) into the target cells. Our data indicated that exosomal THBS2 resides on the surface membrane rather than inside of the secreted exosomes, implying that it is likely to function through directly binding to the membrane protein of its target cells, which is similar to that of non-exosomal THBS2. Also, the above three ways might occur simultaneously. As such, it remains to define whether the released contents contained in exosomes, e.g. DNA, RNA, or other proteins beyond THBS2, exert effects on the target cells. Specifically and selectively delivering THBS2 by means of engineering exosomes may provide valuable insights 91. Collectively, our results indicate THBS2 is likely to function through exosomal and non-exosomal forms, and whether these two forms of THBS2 play different roles in promoting the progression of LUAD requires systematic investigations.

Of note, CD36 and CD47 are two well-characterized receptors of THBS2 80. Recent evidence demonstrated that CD36 was selectively upregulated in intratumoral Treg cells 81 and also promoted intratumoral CD8+ T cell dysfunction 82. Thus, targeting CD36 could greatly enhance antitumor responses with anti-PD-1 therapy 81, 82. CD47, a 'marker-of-self' protein that is broadly overexpressed across tumor cells, is also emerging as a novel macrophage immune checkpoint for cancer immunotherapy 83. Interestingly, our mIHC data showed that THBS2 potentially predominantly affected B, CD8+ T cells and macrophages, and THBS2 expression was inversely associated with CD8+ T cell infiltrates (**Figure 7F-H; Figure 8; Figure S14**). Functionally, THBS2 suppressed the proliferation of CD3+ T cells infiltrates (**Figure 10B**), which in line with a previous study showing that the peptide 4N1K, conserved in all thrombospondin isoforms and mimics the activity of the COOH-terminal cell-binding domain, induces the death of activated, but not resting T cells, via a CD47-dependent mechanism 92. Nevertheless, detailed mechanistic insights into how THBS2+ CAFs shape tumor immunity and modulate the immunotherapy response warrant further study. Intriguingly, recent evidence shows that TGF-β1-THBS2 feedback circuit plays a key role in promoting the progression of pancreatic ductal adenocarcinoma (PDAC) 93. Mechanistically, cancer cell-secreted TGF-β1 activated CAFs to induce THBS2 expression through the Smad2/3 pathway. Then, CAF-derived THBS2 binds to its cognate receptors integrin αvβ3/CD36, leading to the activation of MAPK pathway to promote tumor growth.

## Limitations

There are some limitations of this study. For instance, there is a lack of a large and independent LUAD cohort to validate exosomal THBS2 as an effective biomarker to define high-risk early-stage LUAD populations**.** Likewise, the sample size for the multiplexed IHC investigating the association between THBS2 and immune cell infiltrates was very small, thus requiring an extended sample size for further validation. Furthermore, a direct validation with CAFs-derived THBS2 by culturing and purifying clinical LUAD samples is also needed. Also, we did not explore the role of exosomal THBS2 in the progress of LUAD, which requires systematic investigations in the future.

## Conclusions

Overall, we uncovered a biomarker THBS2, produced by a subset of tumor-specific THBS2+ CAF subpopulation, to stratify a subgroup of early-stage LUAD patients. THBS2 might play a pleiotropic role in modulating cancer cells and particularly tumor immunity. Our study provides not only a biomarker for predicting clinical outcomes of pN0-stage LUAD but also a potential target for therapeutic intervention.

## Supplementary Material

Supplementary figures.

## Abbreviations

CAF: cancer-associated fibroblast
ECM: extracellular matrix
EGFR-TKIs: epidermal growth factor receptor tyrosine kinase inhibitors
GSEA: gene set enrichment analysis
IHC: Immunohistochemistry
LUAD: lung adenocarcinoma
LUSC: lung squamous cell carcinoma
mIHC: Multiplexed IHC
NTA: nanoparticle tracking analysis
OS: overall survival
RFS: recurrence-free survival
scRNA-seq: single-cell RNA sequencing
TCGA: The Cancer Genome Atlas
TEM: transmission electron microscopy
TIME: tumor immune microenvironment
TME: tumor microenvironment
WGCNA: weighted gene co-expression analysis
