# Supplementary material for: Multi-scale integrative analyses identify THBS2+ cancer-associated fibroblasts as a key orchestrator promoting aggressiveness in early-stage lung adenocarcinoma
Source: Theranostics. 2022 Mar 28;12(7):3104–30. doi: 10.7150/thno.69590 (PMC9065207; doi:10.7150/thno.69590)
Supplement: Supplementary file 1 — Supplementary figures. [file thnov12p3104s1.pdf]

Figure S1

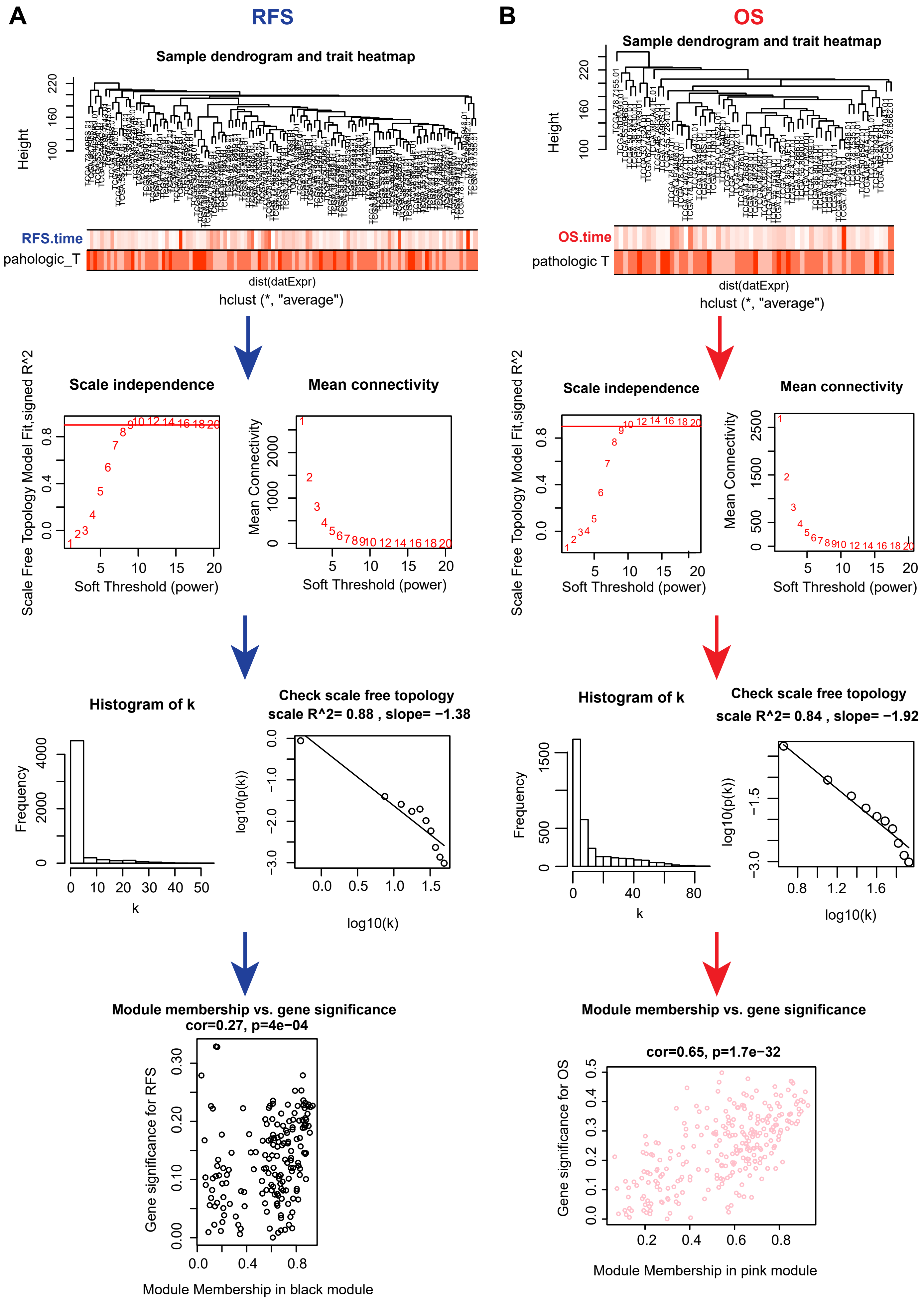
A
RFS
Sample dendrogram and trait heatmap
Height
RFS.time
pahologic_T
dist(datExpr)
hclust (*, "average")
Scale independence
Scale Free Topology Model Fit,signed R^2
Soft Threshold (power)
Mean connectivity
Mean Connectivity
Soft Threshold (power)
Histogram of k
Frequency
k
Check scale free topology
scale R^2= 0.88 , slope= −1.38
log10(p(k))
log10(k)
Module membership vs. gene significance
cor=0.27, p=4e−04
Gene significance for RFS
Module Membership in black module
B
OS
Sample dendrogram and trait heatmap
Height
OS.time
pathologic T
dist(datExpr)
hclust (*, "average")
Scale independence
Scale Free Topology Model Fit,signed R^2
Soft Threshold (power)
Mean connectivity
Mean Connectivity
Soft Threshold (power)
Histogram of k
Frequency
k
Check scale free topology
scale R^2= 0.84 , slope= −1.92
log10(p(k))
log10(k)
Module membership vs. gene significance
cor=0.65, p=1.7e−32
Gene significance for OS
Module Membership in pink module

Figure S1. WGCNA analysis showing the gene modules predicting RFS and OS in pN0-stage LUAD

A, B, WGCNA analysis of gene expression generated by unsupervised hierarchical clustering. The upper panel shows the sample dendrogram and trait heatmap. The color row underneath the dendrogram shows the module assignment determined by the dynamic tree cut. The middle panel shows a histogram of network connectivity and log-log plot of the same histogram. The bottom panel shows the correlation between GS (gene signature, correlation between gene significance) and MM (molecular membership, module membership degree) in black and pink modules.

Figure S2

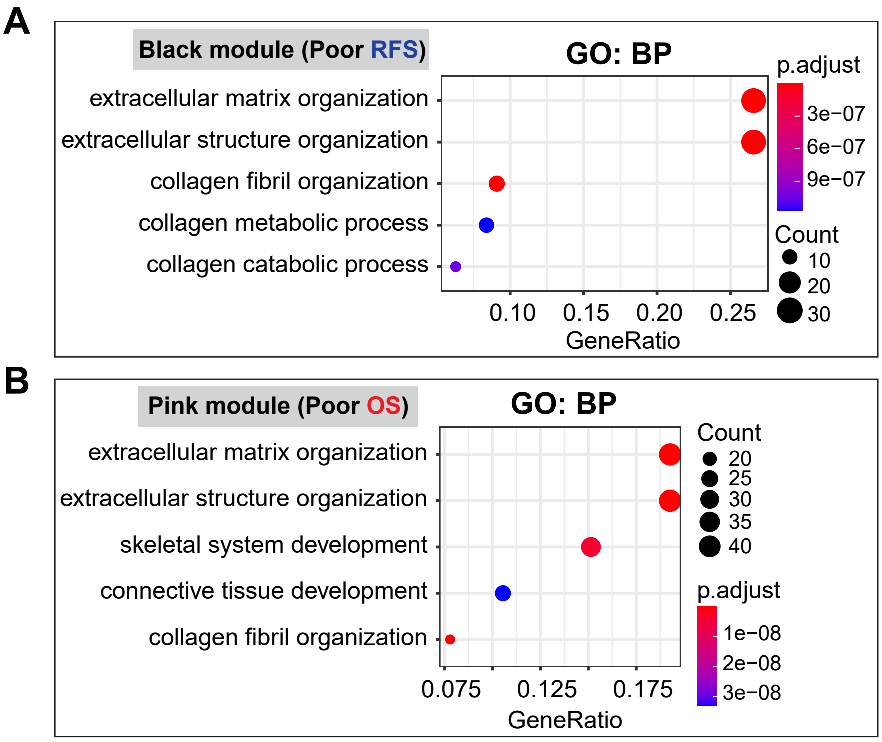

Figure S2. Pathway enrichment analysis of genes in yellow and brown modules

A, B, GO-BP (Gene Ontology Biological Processes) enrichment analyses of genes in black (related to RFS; A) and pink (related to OS; B) modules.

Figure S3

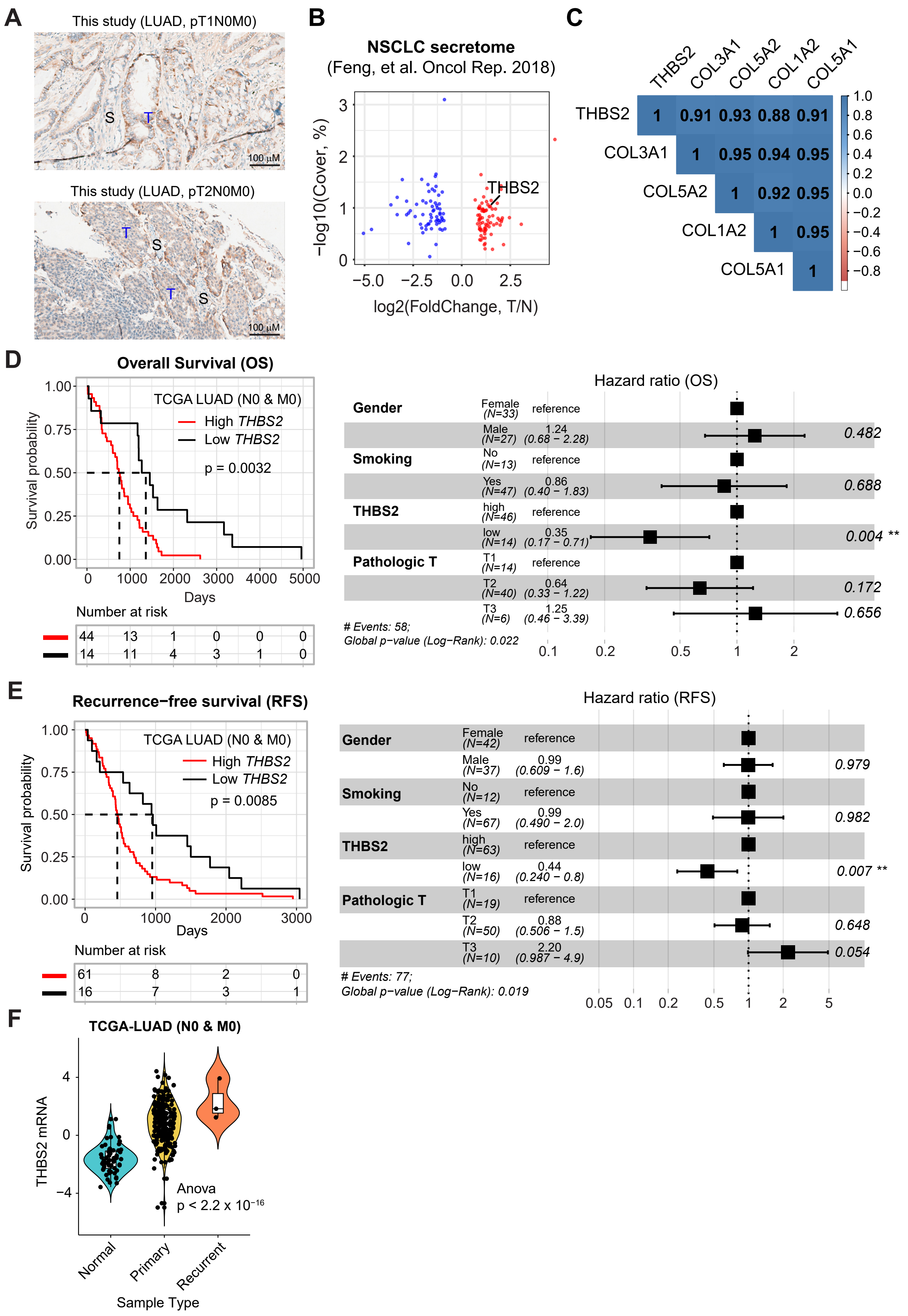
A
This study (LUAD, pT1N0M0)
100 µM
This study (LUAD, pT2N0M0)
100 µM
B
NSCLC secretome
(Feng, et al. Oncol Rep. 2018)
THBS2
−log10(Cover, %)
log2(FoldChange, T/N)
C
THBS2 COL3A1 COL5A2 COL1A2 COL5A1
THBS2 1 0.91 0.93 0.88 0.91
COL3A1 1 0.95 0.94 0.95
COL5A2 1 0.92 0.95
COL1A2 1 0.95
COL5A1 1
D
Overall Survival (OS)
TCGA LUAD (N0 & M0)
High THBS2
Low THBS2
p = 0.0032
Survival probability
Days
Number at risk
44 13 1 0 0 0
14 11 4 3 1 0
Hazard ratio (OS)
Gender Female (N=33) reference
Male (N=27) 1.24 (0.68 − 2.28) 0.482
Smoking No (N=13) reference
Yes (N=47) 0.86 (0.40 − 1.83) 0.688
THBS2 high (N=46) reference
low (N=14) 0.35 (0.17 − 0.71) 0.004 **
Pathologic T T1 (N=14) reference
T2 (N=40) 0.64 (0.33 − 1.22) 0.172
T3 (N=6) 1.25 (0.46 − 3.39) 0.656
Events: 58; Global p−value (Log−Rank): 0.022
E
Recurrence−free survival (RFS)
TCGA LUAD (N0 & M0)
High THBS2
Low THBS2
p = 0.0085
Survival probability
Days
Number at risk
61 8 2 0
16 7 3 1
Hazard ratio (RFS)
Gender Female (N=42) reference
Male (N=37) 0.99 (0.609 − 1.6) 0.979
Smoking No (N=12) reference
Yes (N=67) 0.99 (0.490 − 2.0) 0.982
THBS2 high (N=63) reference
low (N=16) 0.44 (0.240 − 0.8) 0.007 **
Pathologic T T1 (N=19) reference
T2 (N=50) 0.88 (0.506 − 1.5) 0.648
T3 (N=10) 2.20 (0.987 − 4.9) 0.054
Events: 77; Global p−value (Log−Rank): 0.019
F
TCGA-LUAD (N0 & M0)
THBS2 mRNA
Anova
p < 2.2 x 10−16
Normal Primary Recurrent
Sample Type

Figure S3. THBS2 predicts the prognosis of pN0-stage LUAD

A, Representative images showing the location and expression of THBS2 in early-stage LUAD.

B, Mining public secretome profiling of non-small cell lung cancer (NSCLC) samples. Red dots indicate the significantly ($p < 0.05$) upregulated secreted proteins from the tissue culture medium of lung cancer patients compared with matched normal lung tissue. Of note, THBS2 is highly secreted by NSCLC tumors.

C, Correlation matrix showing the correlations among the top 5 genes (THBS2, COL3A1, COL5A2, COL1A2, COL5A1) that were predictive RFS and OS (related to Figure 2G).

D, E, Univariate (left) and multivariate (right, forest plot) analyses showing the association of THBS2 expression with OS (D) and RFS (E) in patients with pN0-stage LUAD.

F, THBS2 gene expression across TCGA pN0-stage primary, recurrent LUAD and normal lung tissue.

Figure S4

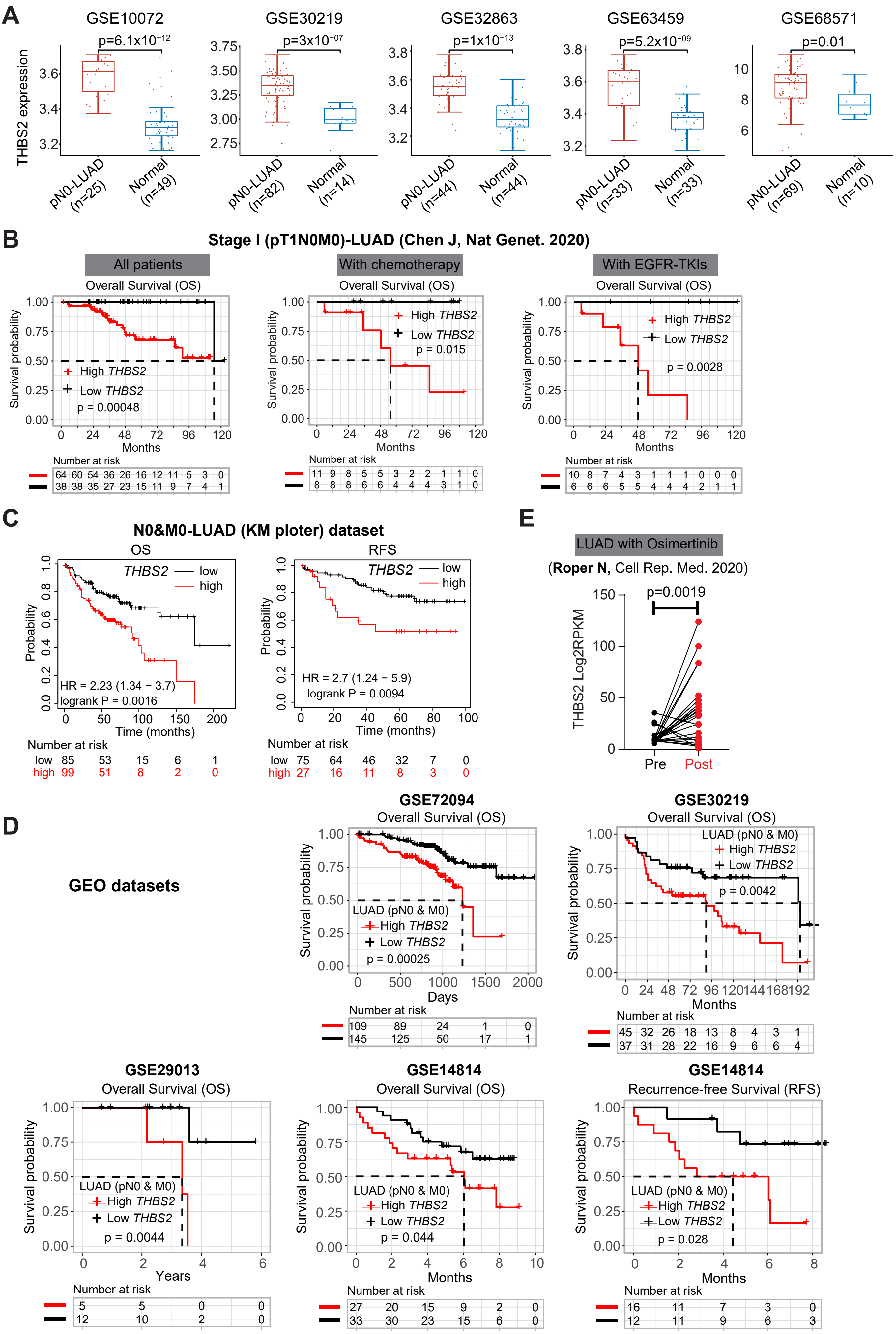
A
GSE10072
p=6.1x10^-12
GSE30219
p=3x10^-07
GSE32863
p=1x10^-13
GSE63459
p=5.2x10^-09
GSE68571
p=0.01
THBS2 expression
pN0-LUAD (n=25)
Normal (n=49)
pN0-LUAD (n=82)
Normal (n=14)
pN0-LUAD (n=44)
Normal (n=44)
pN0-LUAD (n=33)
Normal (n=33)
pN0-LUAD (n=69)
Normal (n=10)
B
Stage I (pT1N0M0)-LUAD (Chen J, Nat Genet. 2020)
All patients
With chemotherapy
With EGFR-TKIs
Overall Survival (OS)
Survival probability
Months
High THBS2
Low THBS2
p = 0.00048
p = 0.015
p = 0.0028
Number at risk
64 60 54 36 26 16 12 11 5 3 0
38 38 35 27 23 15 11 9 7 4 1
11 9 8 5 5 3 2 2 1 1 0
8 8 8 6 6 4 4 4 3 1 0
10 8 7 4 3 1 1 1 0 0 0
6 6 6 5 5 4 4 4 2 1 1
C
N0&M0-LUAD (KM ploter) dataset
OS
RFS
THBS2 low high
Probability
HR = 2.23 (1.34 − 3.7)
logrank P = 0.0016
HR = 2.7 (1.24 − 5.9)
logrank P = 0.0094
Time (months)
Number at risk
low 85 53 15 6 1
high 99 51 8 2 0
low 75 64 46 32 7 0
high 27 16 11 8 3 0
E
LUAD with Osimertinib
(Roper N, Cell Rep. Med. 2020)
p=0.0019
THBS2 Log2RPKM
Pre
Post
D
GEO datasets
GSE72094
Overall Survival (OS)
LUAD (pN0 & M0)
High THBS2
Low THBS2
p = 0.00025
Days
Number at risk
109 89 24 1 0
145 125 50 17 1
GSE30219
Overall Survival (OS)
LUAD (pN0 & M0)
High THBS2
Low THBS2
p = 0.0042
Months
Number at risk
45 32 26 18 13 8 4 3 1
37 31 28 22 16 9 6 6 4
GSE29013
Overall Survival (OS)
LUAD (pN0 & M0)
High THBS2
Low THBS2
p = 0.0044
Years
Number at risk
5 5 0 0
12 10 2 0
GSE14814
Overall Survival (OS)
LUAD (pN0 & M0)
High THBS2
Low THBS2
p = 0.044
Months
Number at risk
27 20 15 9 2 0
33 30 23 15 6 0
GSE14814
Recurrence-free Survival (RFS)
LUAD (pN0 & M0)
High THBS2
Low THBS2
p = 0.028
Months
Number at risk
16 11 7 3 0
12 11 9 6 3

Figure S4. Cross-validation of THBS2 as a prognostic biomarker in early pN0-stage LUAD

A, THBS2 gene expression across pN0M0-stage LUAD and adjacent normal lung tissue from multiple public cohorts. B-D, The association of THBS2 gene expression with the survival of pN0M0-stage LUAD across multiple public cohorts. In B, data were downloaded from Chen J, Nat Genet. 2020; in C, data were downloaded from the Kaplan-Meier plotter portal (Kaplan-Meier plotter [Lung] (kmplot.com)); in D, data were downloaded from GEO datasets (https://www.ncbi.nlm.nih.gov/geo/). Of note, in B, pN0M0-stage LUAD patients who received adjuvant chemotherapy (middle) or EGFR-TKI targeted therapy (right) were specifically investigated. In addition, of the 5 datasets shown in Fig. S4A, there were only transcriptomic data of early-stage LUAD and matched normal lung but without the survival data in GSE10072 and GSE32863. Although the survival data in GSE63459 were available, the cancer-related death only occurred in 5 patients in the entire cohort, leading to the fact that there are no sufficient endpoint events for analysis. For GSE68571, the survival data were provided, but our analysis (not shown) suggested a dramatical survival difference between the THBS2-high vs. THBS2-low pN0-stage LUAD, although the significance was not reached, which is largely due to the short follow-up in this study cohort because the median survival in the two subgroups was not reached.

E, Differentially THBS2 expression (log2RPKM [Reads Per Kilobase per Million mapped reads]) in *EGFR*-mutant LUAD tumors with pre- and post-osimertinib (a third-generation of epidermal growth factor receptor (EGFR)-tyrosine kinase inhibitor (TKI)) treatment. Transcriptomic data were downloaded from Roper N, Cell Rep. Med. 2020. P-value was calculated by paired student`s t-test.

Figure 5

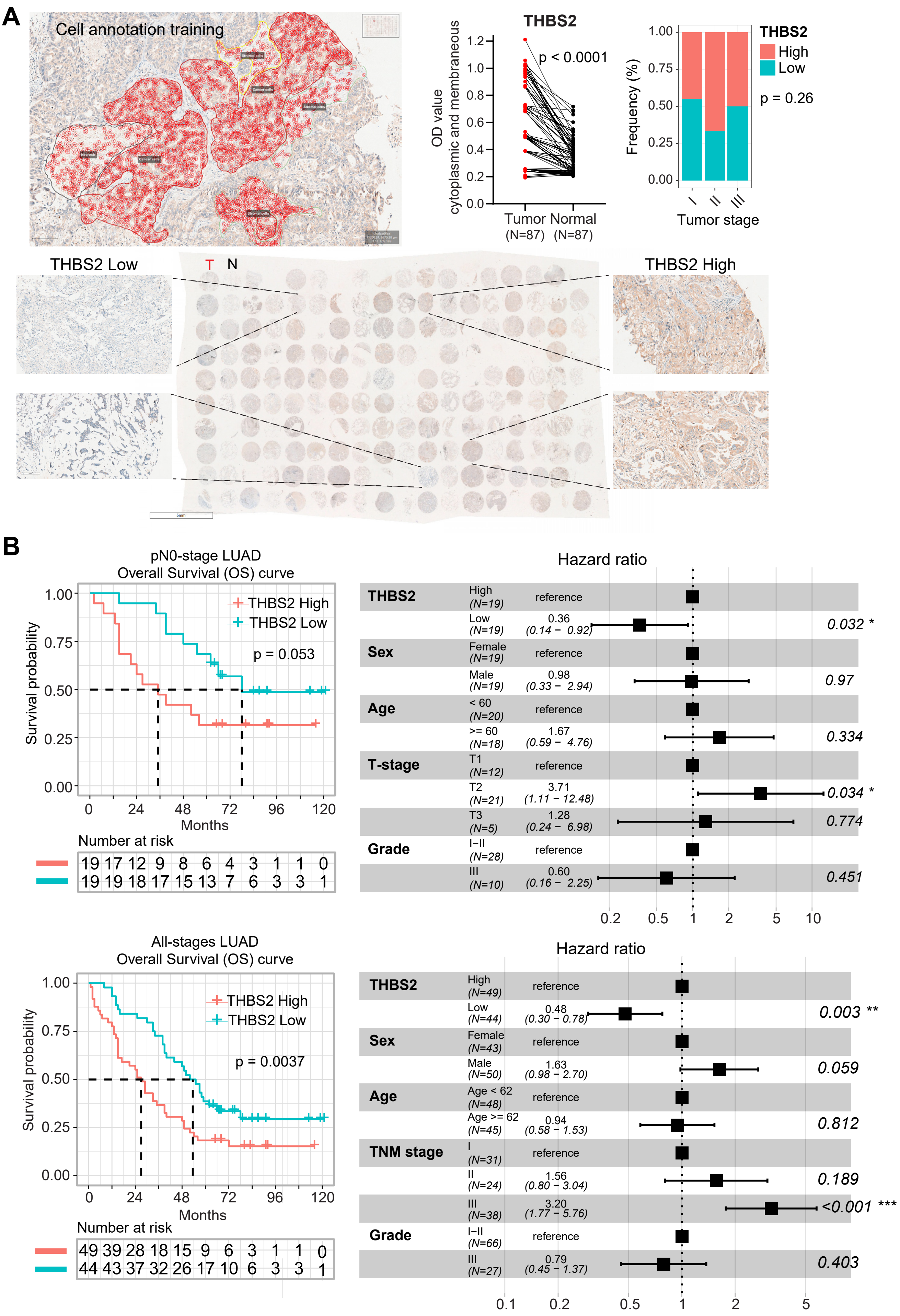

A
Cell annotation training
THBS2
p < 0.0001
OD value cytoplasmic and membraneous
Tumor Normal
(N=87) (N=87)
THBS2
High
Low
p = 0.26
Frequency (%)
I II III
Tumor stage
THBS2 Low
T N
THBS2 High
B
pN0-stage LUAD
Overall Survival (OS) curve
THBS2 High
THBS2 Low
p = 0.053
Survival probability
Months
Number at risk
19 17 12 9 8 6 4 3 1 1 0
19 19 18 17 15 13 7 6 3 3 1
Hazard ratio
THBS2 High (N=19) reference
Low (N=19) 0.36 (0.14 − 0.92) 0.032 *
Sex Female (N=19) reference
Male (N=19) 0.98 (0.33 − 2.94) 0.97
Age < 60 (N=20) reference
>= 60 (N=18) 1.67 (0.59 − 4.76) 0.334
T-stage T1 (N=12) reference
T2 (N=21) 3.71 (1.11 − 12.48) 0.034 *
T3 (N=5) 1.28 (0.24 − 6.98) 0.774
Grade I−II (N=28) reference
III (N=10) 0.60 (0.16 − 2.25) 0.451
0.2 0.5 1 2 5 10
All-stages LUAD
Overall Survival (OS) curve
THBS2 High
THBS2 Low
p = 0.0037
Survival probability
Months
Number at risk
49 39 28 18 15 9 6 3 1 1 0
44 43 37 32 26 17 10 6 3 3 1
Hazard ratio
THBS2 High (N=49) reference
Low (N=44) 0.48 (0.30 − 0.78) 0.003 **
Sex Female (N=43) reference
Male (N=50) 1.63 (0.98 − 2.70) 0.059
Age Age < 62 (N=48) reference
Age >= 62 (N=45) 0.94 (0.58 − 1.53) 0.812
TNM stage I (N=31) reference
II (N=24) 1.56 (0.80 − 3.04) 0.189
III (N=38) 3.20 (1.77 − 5.76) <0.001 ***
Grade I−II (N=66) reference
III (N=27) 0.79 (0.45 − 1.37) 0.403
0.1 0.2 0.5 1 2 5

Figure S5. Internal LUAD cohort validating THBS2 as a prognostic biomarker in early pN0-stage LUAD

A, B, Our internal treatment-naïve LUAD cohort (tissue microarray data; N = 93) showing the association of THBS2 protein levels with OS after surgery. A, Data quantifications were performed at the single-cell level using Qupath software. B, Univariate (left) and multivariate (right, forest plot) analyses showing the association of THBS2 expression with OS in patients with pN0-stage LUAD (upper panel) and the entire cohort (lower panel).

Figure S6

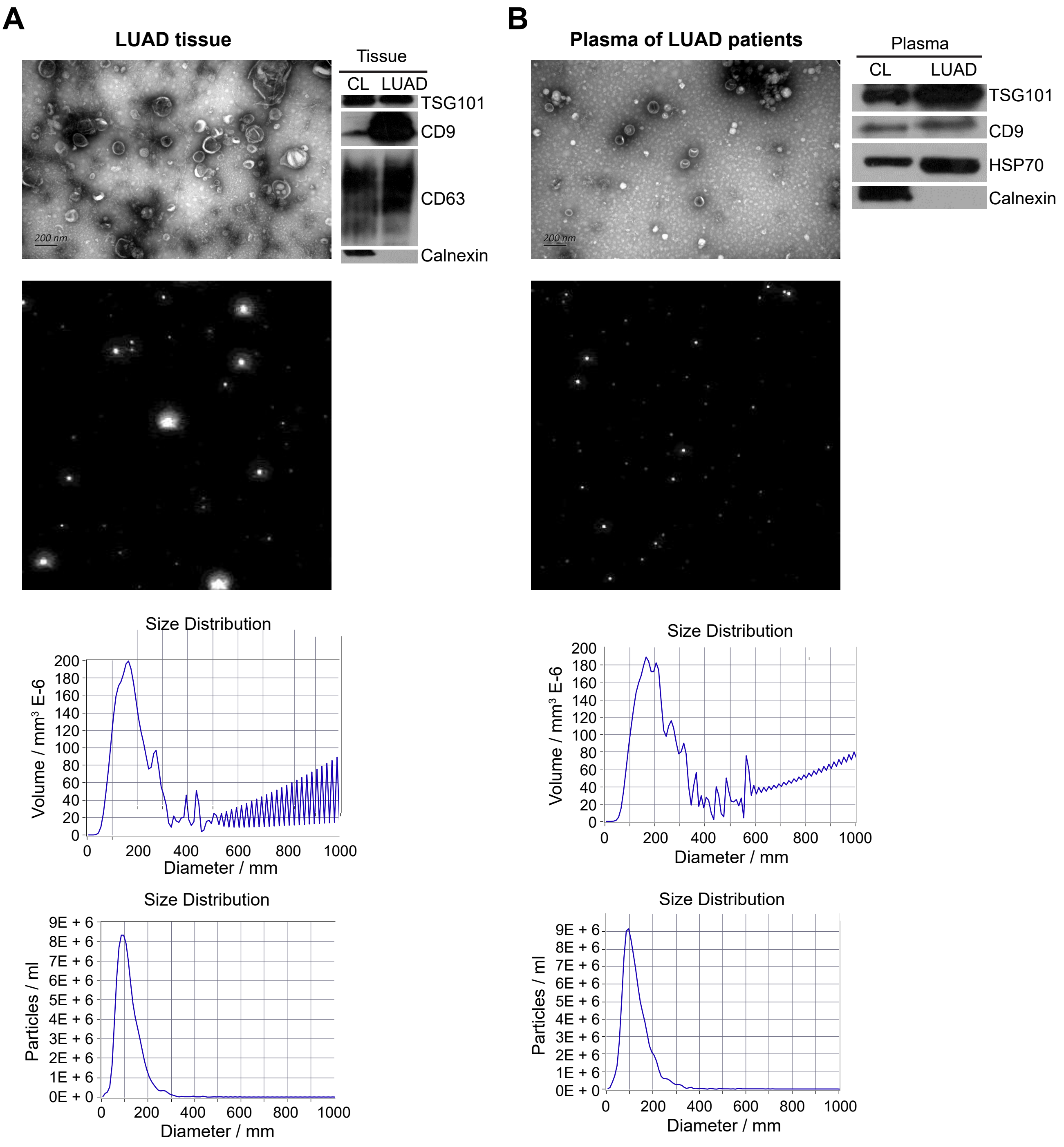

Diameter / nm	Particles / mL	FWHM / nm	Percentage
94.3	8.30E+06	87.1	98.5
402.9	2.40E+04	16.3	0.3
356.6	2.40E+04	10	0.1
514.9	1.10E+04	19.6	0.1
715.3	8.30E+03	30	0

Diameter / nm	Particles/mL	FWHM / nm	Percentage
97.7	9.00E+06	88.5	97.8
412	3.30E+04	25.3	0.1
572.5	3.10E+04	33.1	0.1
614.9	1.50E+04	29.2	0.1
462.4	1.40E+04	100.8	0.2

Fig. S6. Exosome isolation, purification and characterization (related to Figure 4)

A, B, Lung tissue- (A) or plasma (B)-derived exosomes were characterized by transmission electron microscopy (morphology; upper left panel in A, B), and Nanoparticle Tracking Analysis (the size; lower panels in A, B), and western blots using three positive exosomal markers (CD9, TSG101, CD63, or HSP70) and one negative marker (Calnexin) (upper right panel in A, B). CL: cell lysate, used as a positive quanlity control. Scale bar: 200 nm.

Figure S7

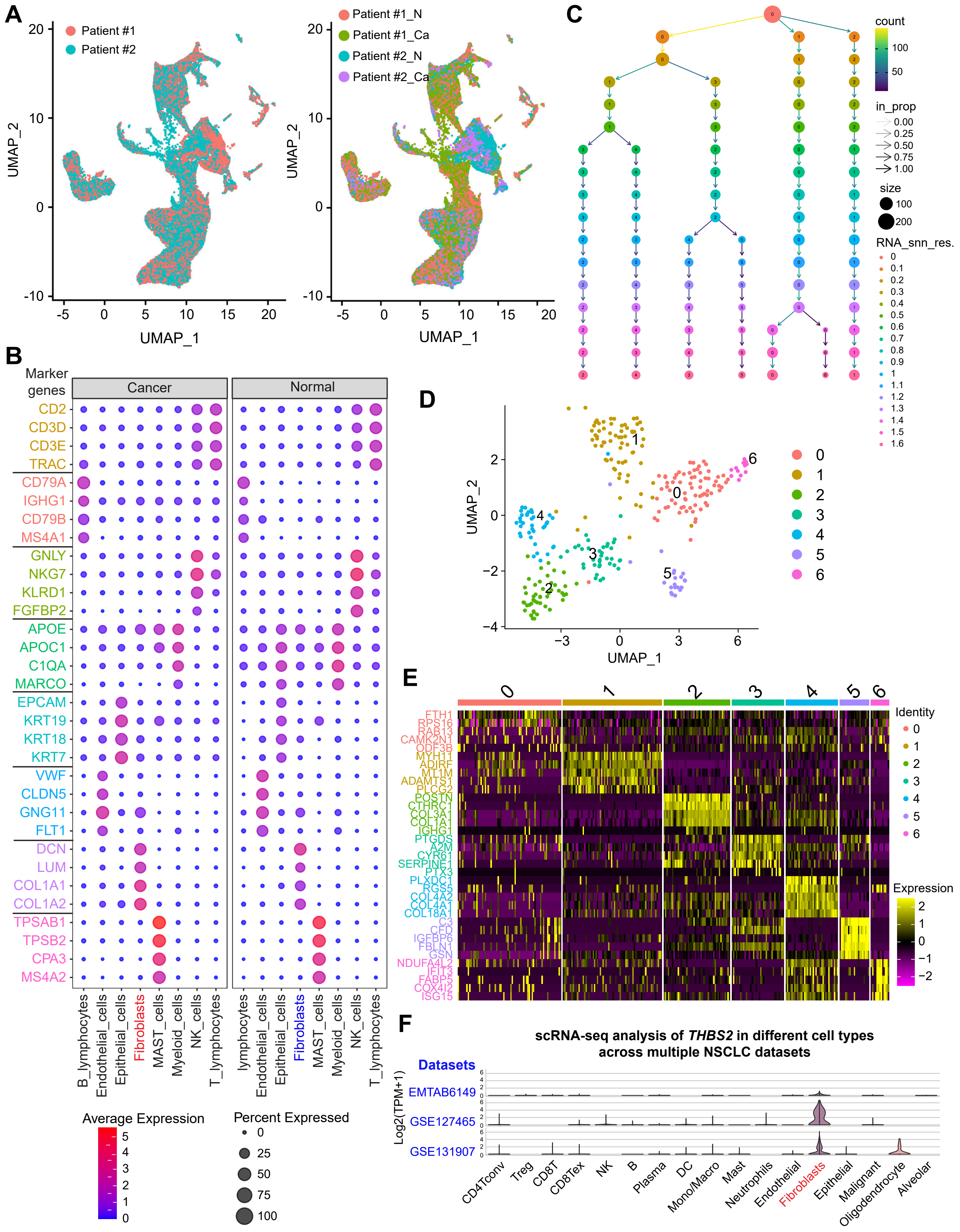
A
Patient #1
Patient #2
Patient #1_N
Patient #1_Ca
Patient #2_N
Patient #2_Ca
UMAP_1
UMAP_2
B
Marker genes
Cancer
Normal
CD2
CD3D
CD3E
TRAC
CD79A
IGHG1
CD79B
MS4A1
GNLY
NKG7
KLRD1
FGFBP2
APOE
APOC1
C1QA
MARCO
EPCAM
KRT19
KRT18
KRT7
VWF
CLDN5
GNG11
FLT1
DCN
LUM
COL1A1
COL1A2
TPSAB1
TPSB2
CPA3
MS4A2
B_lymphocytes
Endothelial_cells
Epithelial_cells
Fibroblasts
MAST_cells
Myeloid_cells
NK_cells
T_lymphocytes
Average Expression
Percent Expressed
C
count
in_prop
size
RNA_snn_res.
D
E
Identity
Expression
FTH1
RPS16
RAB13
CAMK2N1
ODF3B
MYH11
ADIRF
MT1M
ADAMTS1
PLCG2
POSTN
CTHRC1
COL3A1
COL1A1
IGHG1
PTGDS
A2M
CYR61
SERPINE1
PTX3
PLXDC1
RGS5
COL4A2
COL4A1
COL18A1
C3
CFD
IGFBP6
FBLN1
GSN
NDUFA4L2
IFIT3
FABP5
COX4I2
ISG15
F
scRNA-seq analysis of THBS2 in different cell types across multiple NSCLC datasets
Datasets
EMTAB6149
GSE127465
GSE131907
Log2(TPM+1)
CD4Tconv
Treg
CD8T
CD8Tex
NK
B
Plasma
DC
Mono/Macro
Mast
Neutrophils
Endothelial
Fibroblasts
Epithelial
Malignant
Oligodendrocyte
Alveolar

Figure S7. THBS2 defines a unique CAF subset

A, UMAP plots showing patients` origin (left) and samples` origin (right). Related to Figure 5B.

B, Marker genes used to define different cluster identities of whole cell populations. Related to Figure 5B. C, Implementing the Clustree algorithm to determine the optimal cluster separation of CAFs (cancer-associated fibroblasts). R package "Clustree" was used. Related to Figure 5D-G.

D, UMAP plot showing that CAFs could be divided into 7 subclusters, based on C.

E, Heatmap showing the top 5 marker genes of CAFs.

F, Data mining of public NSCLC (non-small cell lung cancer) scRNA-seq data showing THBS2 expression in distinct cell types across different NSCLC datasets. Data were directly obtained from the TISCH portal, a comprehensive web resource enabling interactive single-cell transcriptome visualization of the tumor microenvironment (http://tisch.comp-genomics.org/home/). Of note, THBS2 is consistently expressed by CAFs across these datasets, although CAFs were not sub-grouped here.

Figure S8

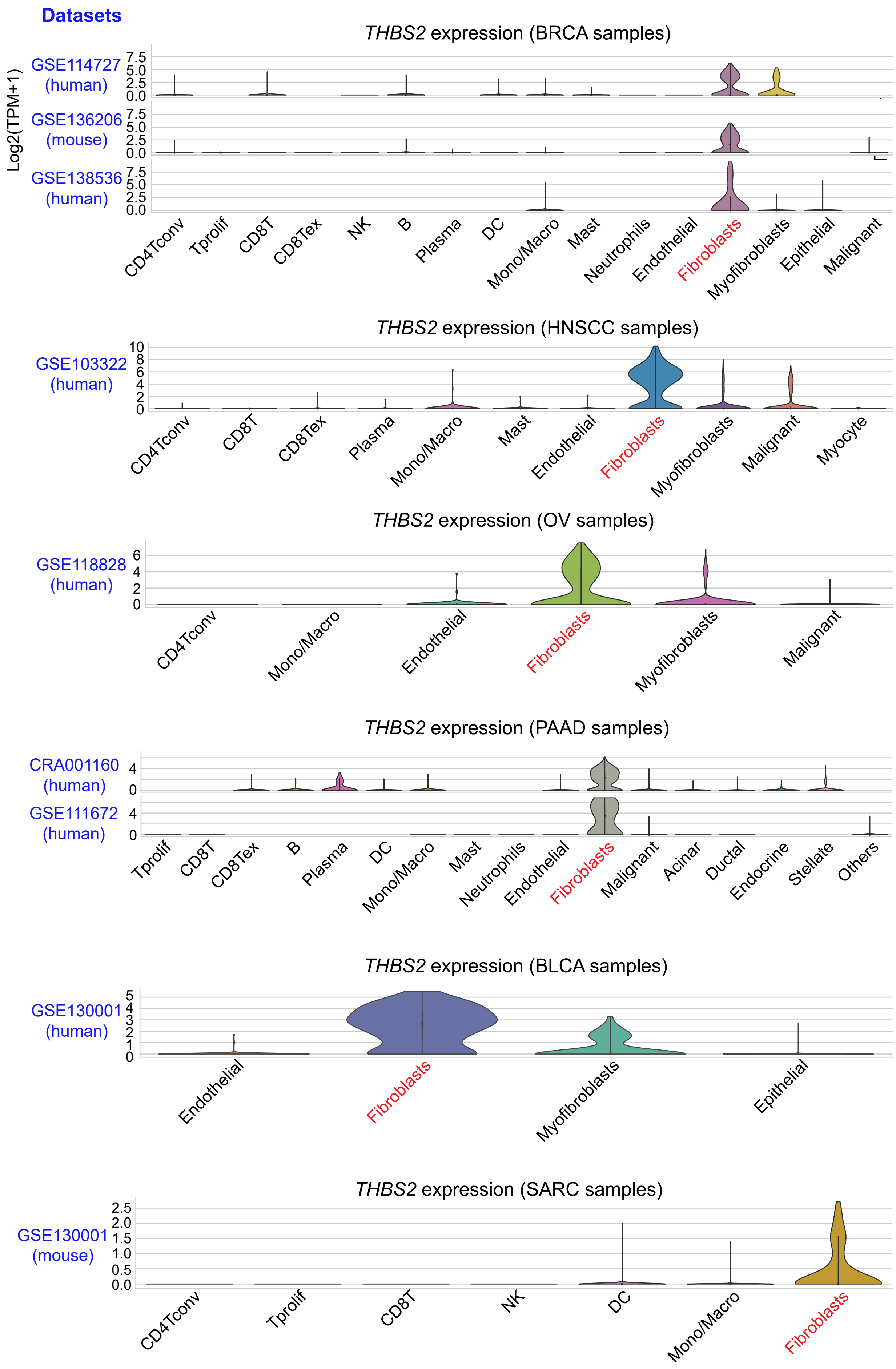

Datasets
THBS2 expression (BRCA samples)
Log2(TPM+1)
GSE114727 (human)
GSE136206 (mouse)
GSE138536 (human)
7.5
5.0
2.5
0.0
CD4Tconv
Tprolif
CD8T
CD8Tex
NK
B
Plasma
DC
Mono/Macro
Mast
Neutrophils
Endothelial
Fibroblasts
Myofibroblasts
Epithelial
Malignant
THBS2 expression (HNSCC samples)
GSE103322 (human)
10
8
6
4
2
0
CD4Tconv
CD8T
CD8Tex
Plasma
Mono/Macro
Mast
Endothelial
Fibroblasts
Myofibroblasts
Malignant
Myocyte
THBS2 expression (OV samples)
GSE118828 (human)
6
4
2
0
CD4Tconv
Mono/Macro
Endothelial
Fibroblasts
Myofibroblasts
Malignant
THBS2 expression (PAAD samples)
CRA001160 (human)
GSE111672 (human)
4
0
Tprolif
CD8T
CD8Tex
B
Plasma
DC
Mono/Macro
Mast
Neutrophils
Endothelial
Fibroblasts
Malignant
Acinar
Ductal
Endocrine
Stellate
Others
THBS2 expression (BLCA samples)
GSE130001 (human)
5
4
3
2
1
0
Endothelial
Fibroblasts
Myofibroblasts
Epithelial
THBS2 expression (SARC samples)
GSE130001 (mouse)
2.5
2.0
1.5
1.0
0.5
0.0
CD4Tconv
Tprolif
CD8T
NK
DC
Mono/Macro
Fibroblasts

Figure S8. Multiple scRNA-seq analyses reveal THBS2 expression predominantly derived from subsets of CAFs

Data mining of public scRNA-seq data showing THBS2 expression in distinct cellular clusters across several cancer types that are enriched for stromal compartments. Data were obtained from the TISCH portal, a comprehensive web resource enabling interactive single-cell transcriptome visualization of the tumor microenvironment.

Figure S9

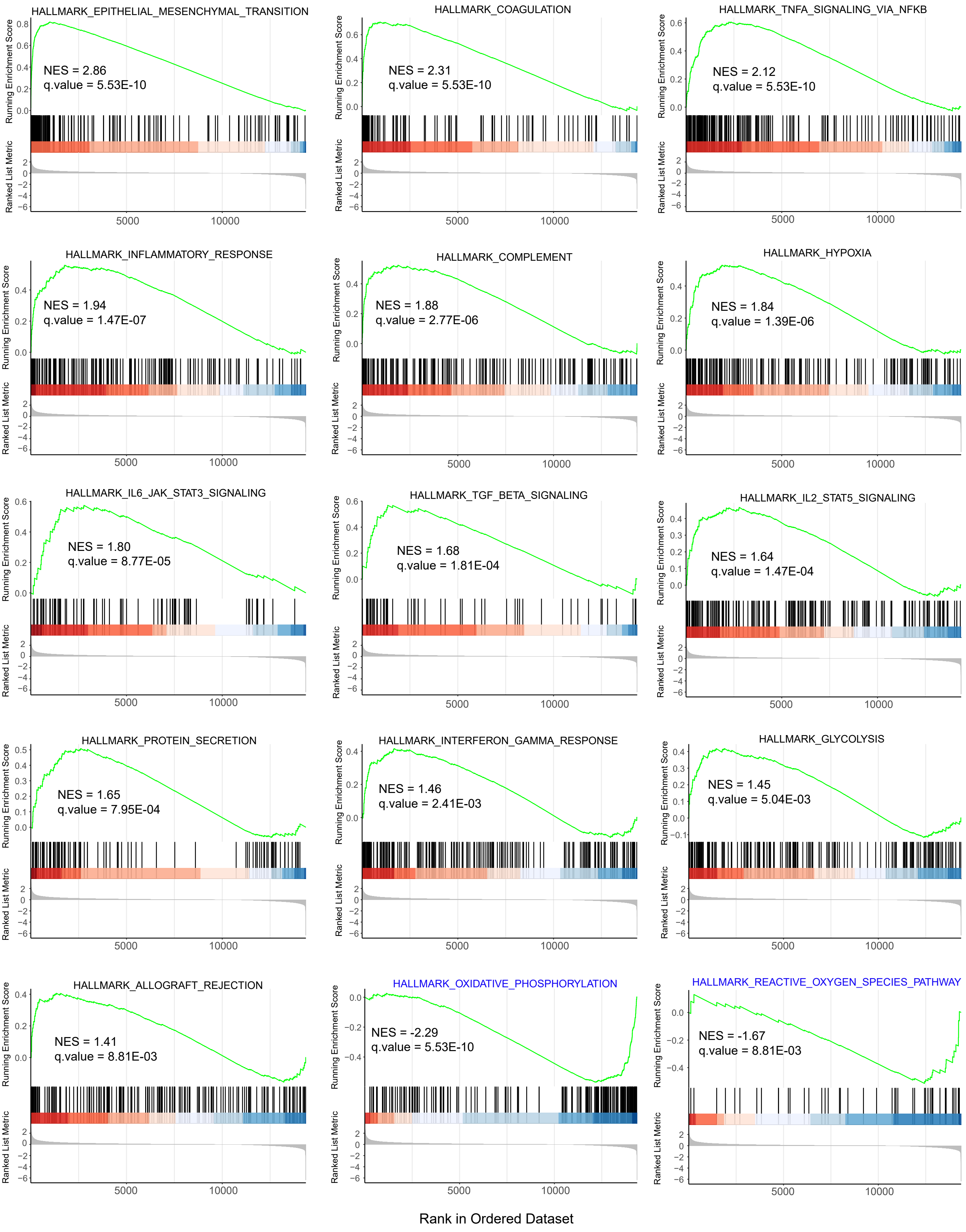

HALLMARK_EPITHELIAL_MESENCHYMAL_TRANSITION
NES = 2.86
q.value = 5.53E-10
HALLMARK_COAGULATION
NES = 2.31
q.value = 5.53E-10
HALLMARK_TNFA_SIGNALING_VIA_NFKB
NES = 2.12
q.value = 5.53E-10
HALLMARK_INFLAMMATORY_RESPONSE
NES = 1.94
q.value = 1.47E-07
HALLMARK_COMPLEMENT
NES = 1.88
q.value = 2.77E-06
HALLMARK_HYPOXIA
NES = 1.84
q.value = 1.39E-06
HALLMARK_IL6_JAK_STAT3_SIGNALING
NES = 1.80
q.value = 8.77E-05
HALLMARK_TGF_BETA_SIGNALING
NES = 1.68
q.value = 1.81E-04
HALLMARK_IL2_STAT5_SIGNALING
NES = 1.64
q.value = 1.47E-04
HALLMARK_PROTEIN_SECRETION
NES = 1.65
q.value = 7.95E-04
HALLMARK_INTERFERON_GAMMA_RESPONSE
NES = 1.46
q.value = 2.41E-03
HALLMARK_GLYCOLYSIS
NES = 1.45
q.value = 5.04E-03
HALLMARK_ALLOGRAFT_REJECTION
NES = 1.41
q.value = 8.81E-03
HALLMARK_OXIDATIVE_PHOSPHORYLATION
NES = -2.29
q.value = 5.53E-10
HALLMARK_REACTIVE_OXYGEN_SPECIES_PATHWAY
NES = -1.67
q.value = 8.81E-03
Running Enrichment Score
Ranked List Metric
5000
10000
Rank in Ordered Dataset

Figure S9. Pathway enrichment analysis of THBS2+ vs. THBS2– CAFs

Gene Set Enrichment Analysis (GSEA) of THBS2+ CAFs compared to THBS2– CAFs based on the scRNA-seq data of this study (related to Figure 5D).

Figure S10

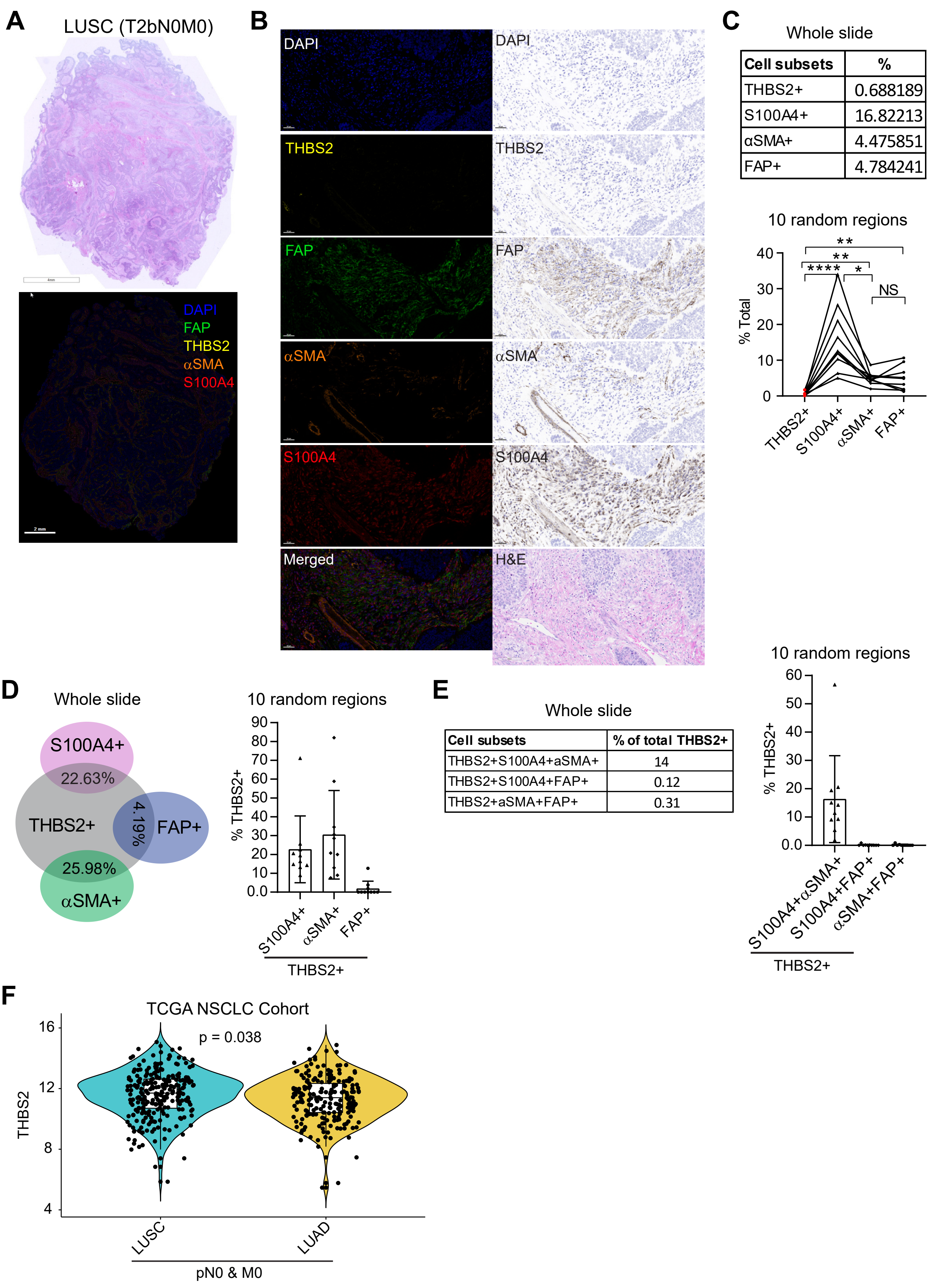

Cell subsets	%
THBS2+	0.688189
S100A4+	16.82213
αSMA+	4.475851
FAP+	4.784241

Cell subsets	% of total THBS2+
THBS2+S100A4+aSMA+	14
THBS2+S100A4+FAP+	0.12
THBS2+aSMA+FAP+	0.31

Figure S10. Multiplexed IHC shows the spatial association between THBS2 and CAF-associated markers in LUSC

A, B, THBS2 co-staining with four classical CAF markers (αSMA, FAP, S100A4 [FSP1]) in a LUSC (lung squamous cell carcinoma) (pT2bN0M0) sample. The image acquisition of all markers occurred simultaneously. Panel A shows the whole slide scan; Scale bar: 2 mm.; panel B shows the representative regions (200x);Scale bar: 50 μm..

C-E, Individual or selected combinations of markers (whole slide and 10 randomly selected regions) were quantified and are shown. *$p < 0.05$; **$p < 0.01$; ****$p < 0.0001$ by paired ANOVA test.

F, Comparative analysis of THBS2 expression between TCGA pN0-stage LUSC and LUAD. P-value was calculated by a two-side student`s t-test.

Figure S11

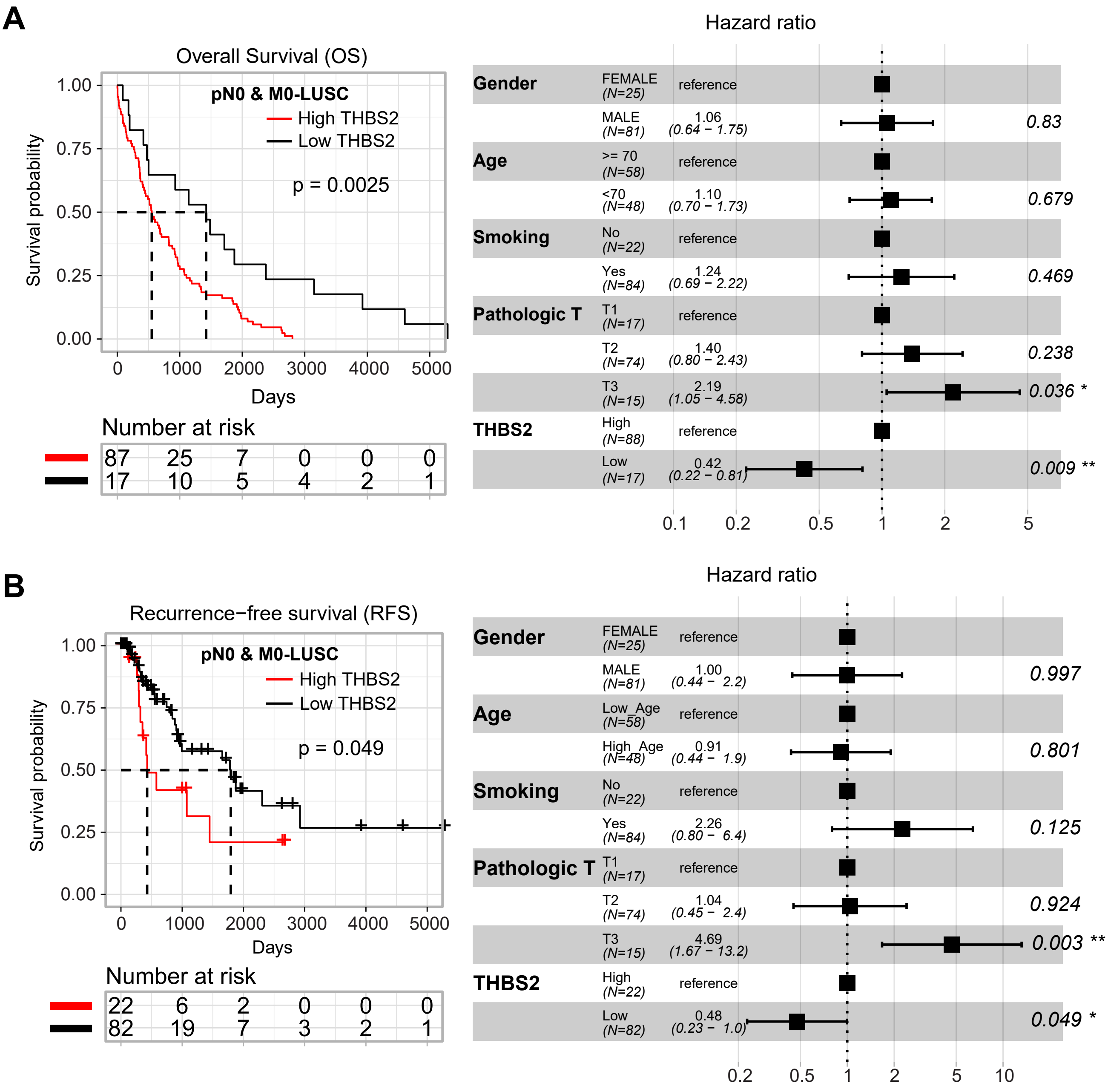

Figure S11. THBS2 expression predicts early pN0-stage LUSC

A, B, Univariate (left) and multivariate (right, forest plot) analyses showing the association of THBS2 expression with OS (A) and RFS (B) in patients with pN0M0-stage LUSC (lung squamous cell carcinoma) after surgery.

Figure S12

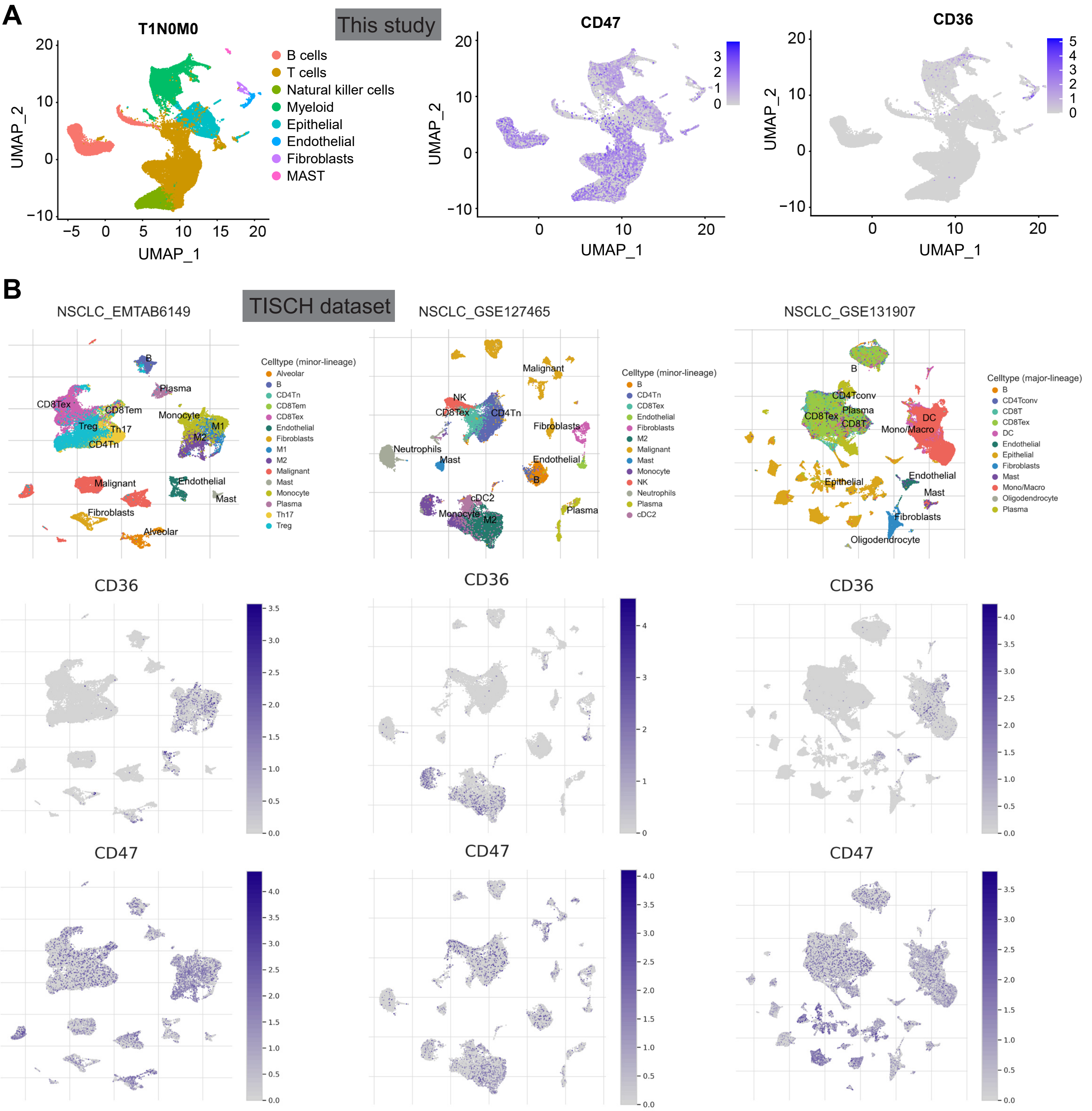

Figure S12. Expression of CD36 and CD47 across cellular subsets of lung tumors

A, B, scRNA-seq showing the gene expression of THBS2 in different cell clusters of lung tumor from this study (LUAD, pT1N0M0; panel A) and public datasets (TISCH portal; panel B).

Figure S13

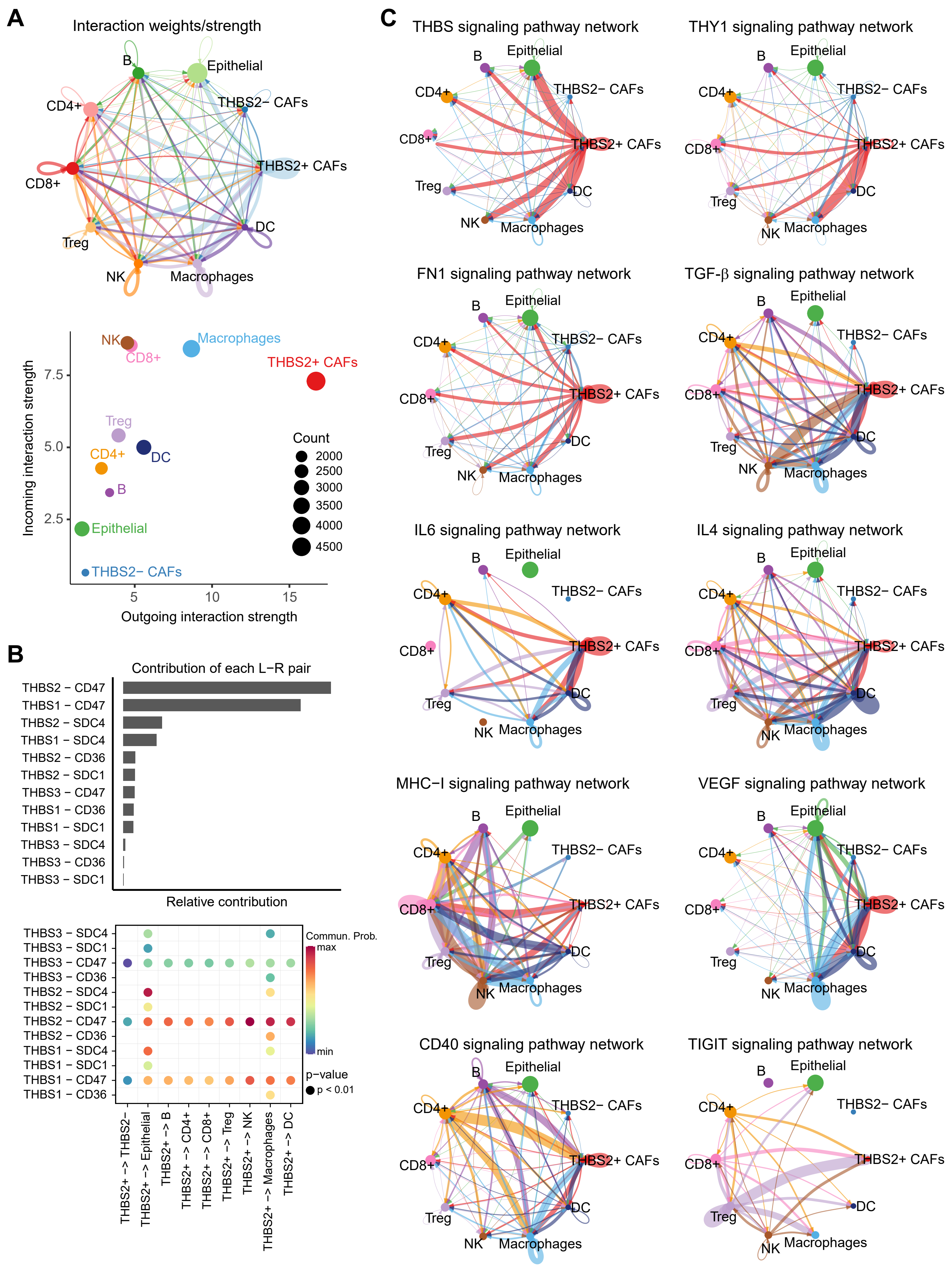
A
Interaction weights/strength
B
Epithelial
CD4+
THBS2− CAFs
THBS2+ CAFs
CD8+
DC
Treg
NK
Macrophages
Incoming interaction strength
Outgoing interaction strength
7.5
5.0
2.5
5
10
15
Count
2000
2500
3000
3500
4000
4500
B
Contribution of each L−R pair
THBS2 − CD47
THBS1 − CD47
THBS2 − SDC4
THBS1 − SDC4
THBS2 − CD36
THBS2 − SDC1
THBS3 − CD47
THBS1 − CD36
THBS1 − SDC1
THBS3 − SDC4
THBS3 − CD36
THBS3 − SDC1
Relative contribution
Commun. Prob.
max
min
p−value
p < 0.01
THBS2+ −> THBS2−
THBS2+ −> Epithelial
THBS2+ −> B
THBS2+ −> CD4+
THBS2+ −> CD8+
THBS2+ −> Treg
THBS2+ −> NK
THBS2+ −> Macrophages
THBS2+ −> DC
C
THBS signaling pathway network
THY1 signaling pathway network
FN1 signaling pathway network
TGF-β signaling pathway network
IL6 signaling pathway network
IL4 signaling pathway network
MHC−I signaling pathway network
VEGF signaling pathway network
CD40 signaling pathway network
TIGIT signaling pathway network

Figure S13. Intercellular communication analysis of THBS2+ CAFs and other cells in early-stage lung cancer

A, Circle plot (top) showing global communications among the major cell types within the microecosystem of lung cancer (pT1N0M0). The edges are directional and the thickness of them corresponds to the signal strength of interactions, colored according to cell type. The size of the node is related to the number of cells in each cell type. Dot plot (bottom) showing the global incoming and outgoing interaction strength of each cell type, indicating the dominant senders and receivers. Data were based on our scRNA-seq of two pT1N0M0 lung cancer samples (related to Figure 4) using CellChat package in R software. Of note, THBS2+ CAFs have more profound interaction weights with both tumor and immune cells compared with THBS2- CAFs.

B, Bar plot (upper panel) showing the relative contribution of each ligand-receptor pair to the THBS signaling pathway. Dot plot (lower planel) showing the potential interaction weights (ligand-receptor pairs) from THBS+ cancer-associated fibroblasts to other cell types.

C, Top enriched intercellular communication networks constructed based on the interactions among ligands, receptors and their cofactors.

Figure S14

A

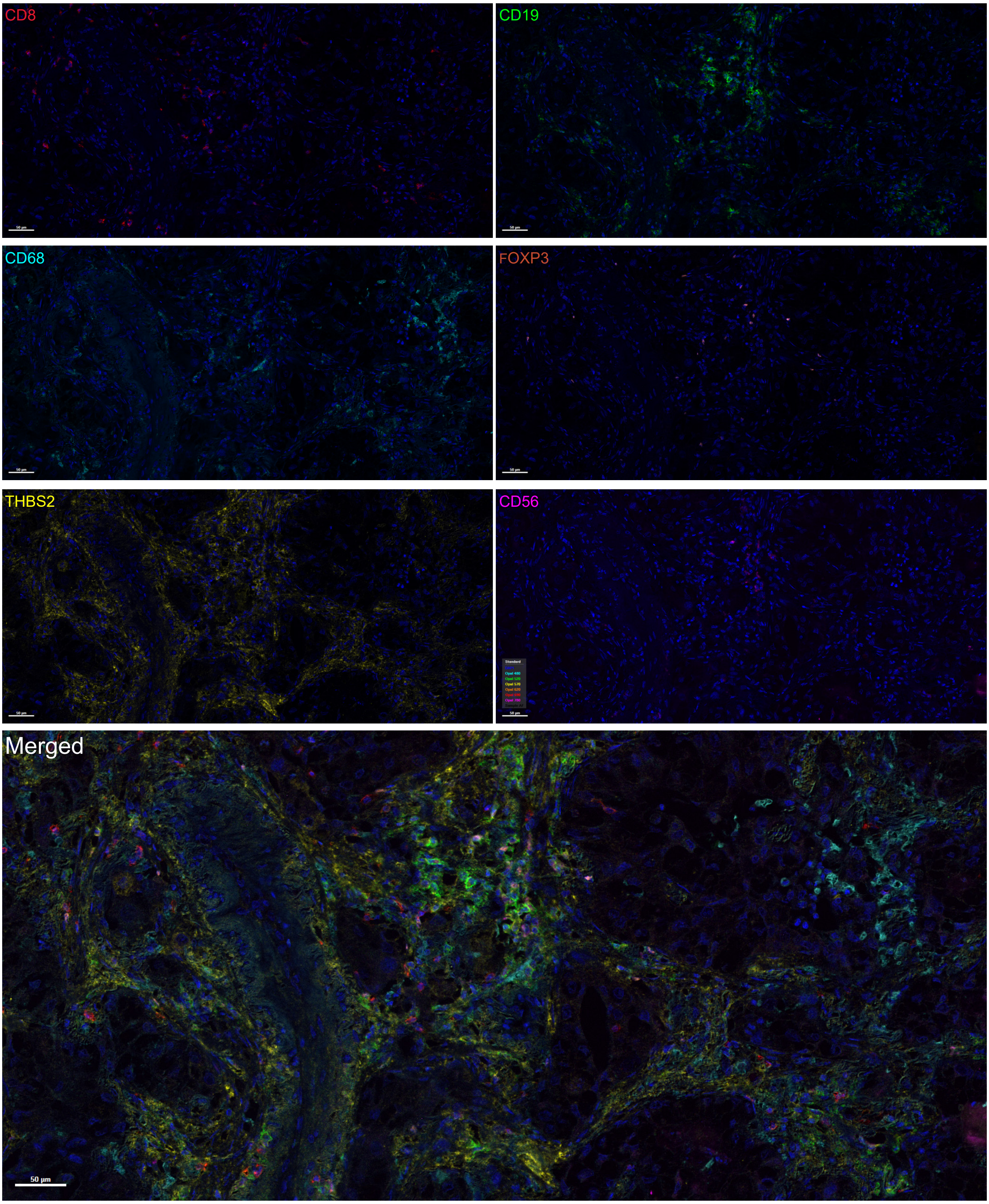

B

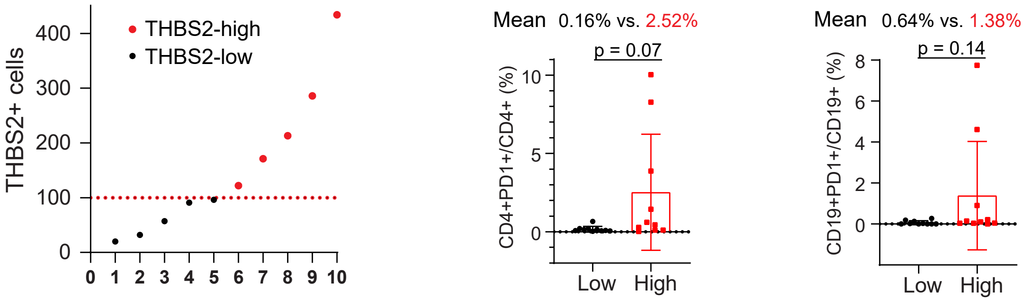

Figure S14. High THBS2 expression is associated with elevated exhaustion markers in immune infiltrates

A, THBS2 co-staining with the indicated immune markers in a resected LUAD (lung adenocarcinoma) (pT2bN0M0) sample. The image acquisition of all markers occurred simultaneously with the representative regions were shown (200x). Scale bar: 50 µm.

B, Ratio of CD4+PD1+ to total CD4+ T cells and CD19+PD1+ to total CD19+ T cells in 10 randomly (5 tumoral and 5 stromal regions) selected regions of a LUAD tumor related to Figure 8. p-value was calculated using a two-side student`s t-test.

Figure S15

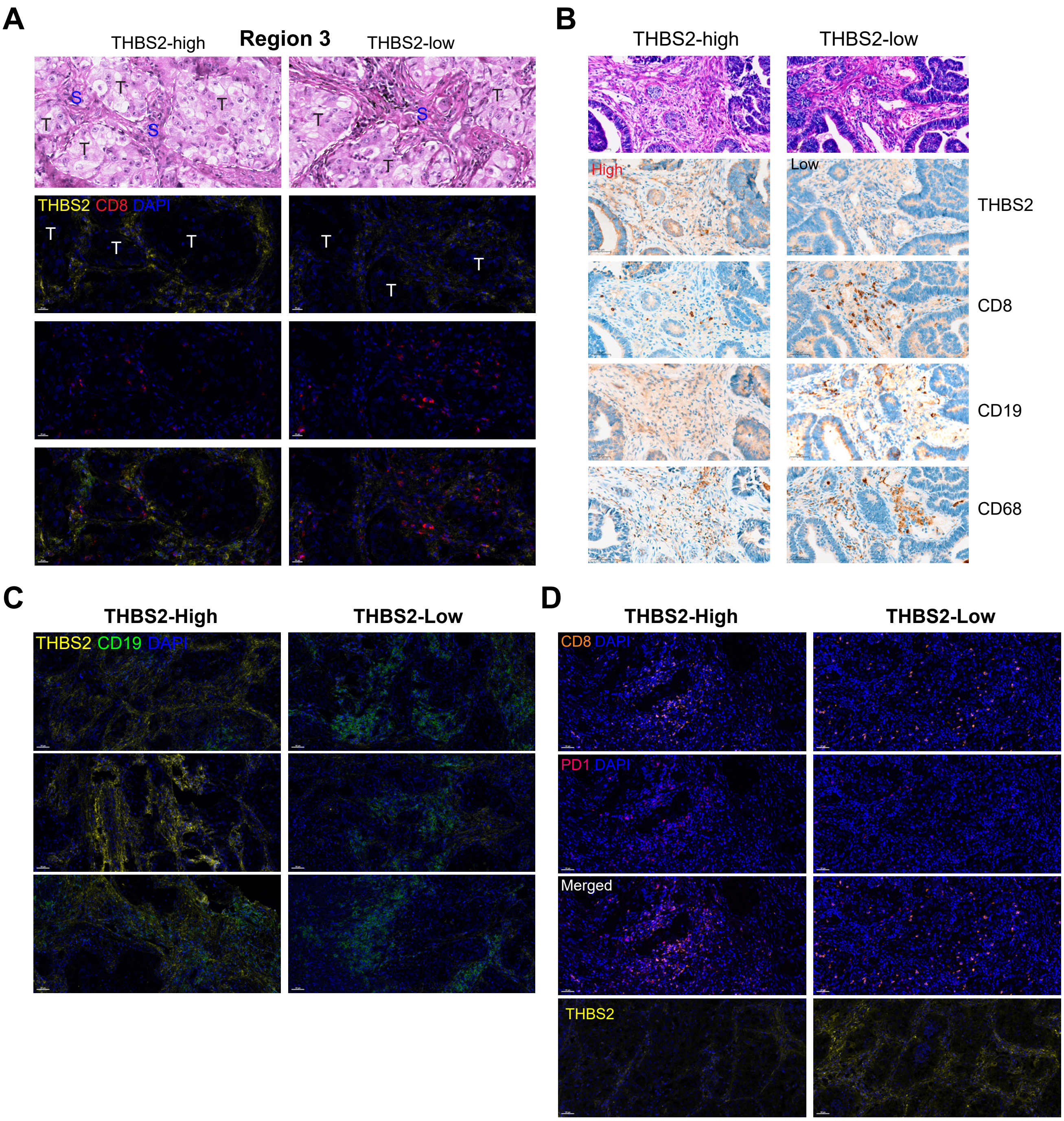

Figure S15. High THBS2 expression is associated with dysregulated immune cell infiltrates.

A, Representative images showing the THBS2 co-staining with CD8 T lymphocytes in another regions of one LUAD sample (related to Figure 8A, B). Scale bar: 50 μm.

B, Representative figures showing the difference in immune cell infiltrates between THBS2-high and -low groups (related to Figure 8C). Scale bar: 50 μm.

C, THBS2 co-staining with the CD19 B lymphocytes in a resected LUAD (lung adenocarcinoma) (pT2bN0M0) sample. The image acquisition of all markers occurred simultaneously with the representative regions were shown (200x; panel A, B). Scale bar: 50 μm.

D, CD8, PD1 expression between between THBS2-high and -low groups (related to Figure 7H). Scale bar: 50 μm.

Figure S16

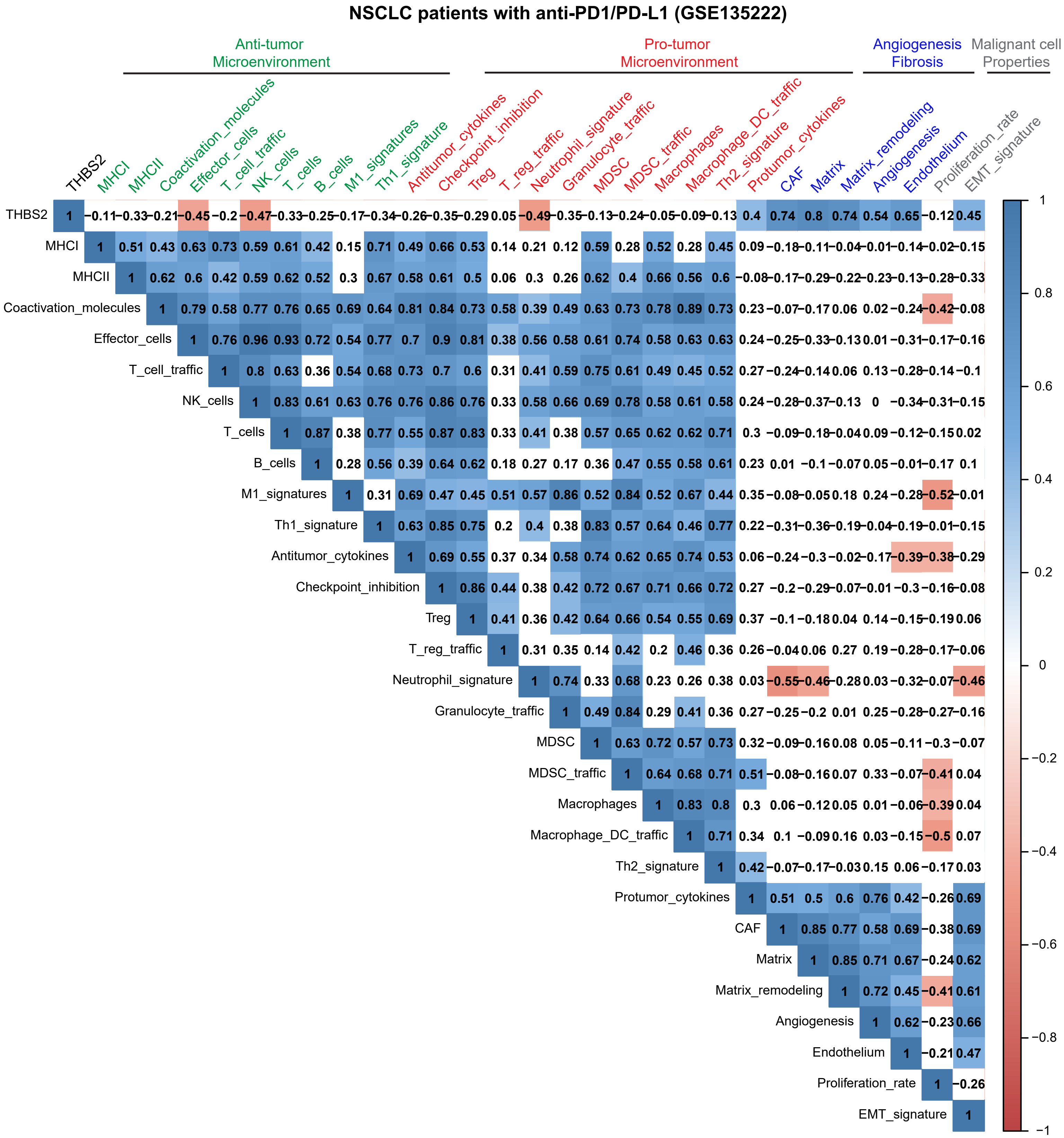

Figure S16. THBS2 is associated with an aberrant tumor immune microenvironment

The correlation matrix showing the correlation between THBS2 expression and the tumor microenvironment signature across NSCLC patients treated with immunotherapy. Data were downloaded and reanalyzed from Bagaev A et al. Cancer Cell. 2021.

Figure S17

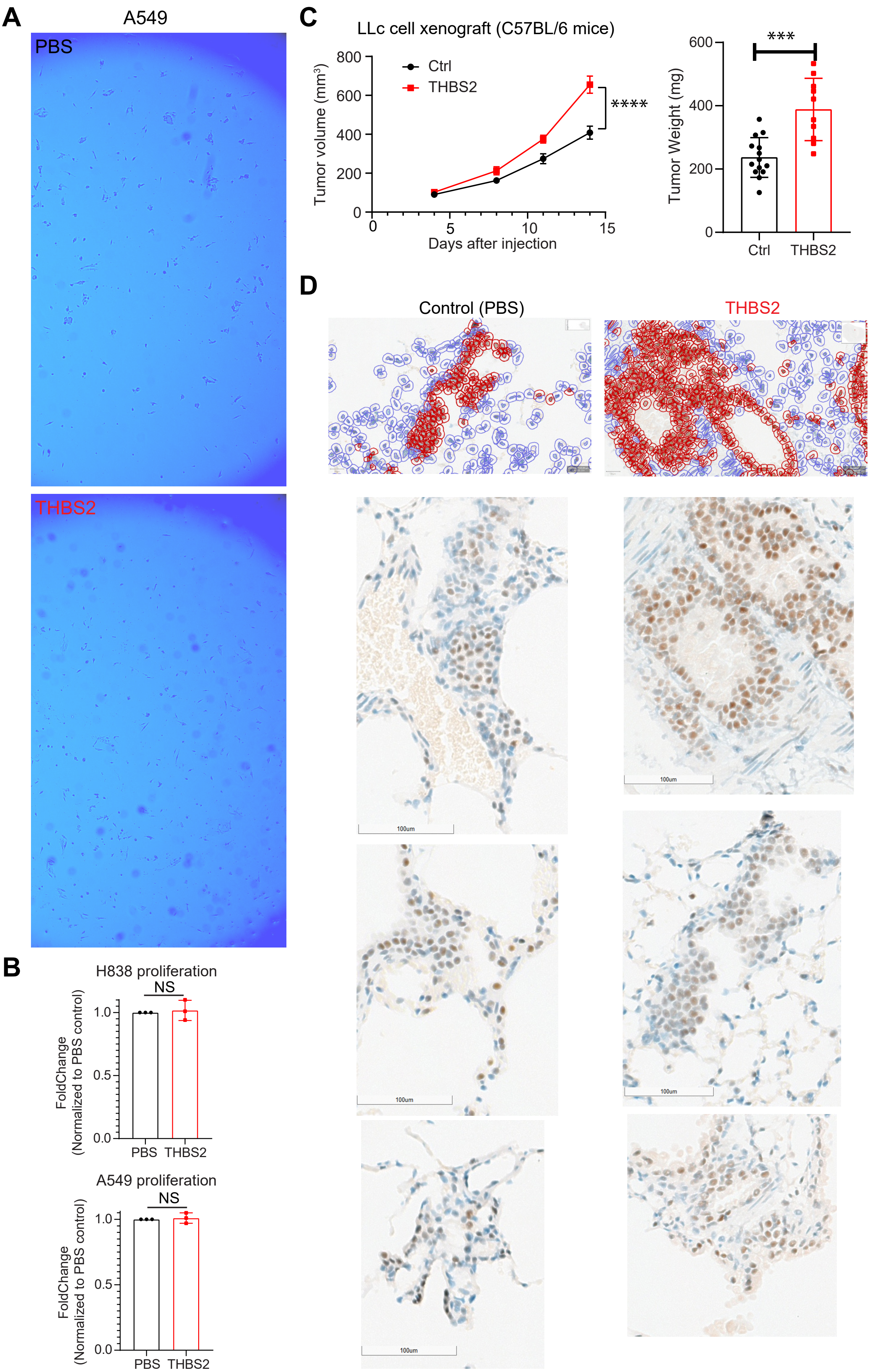
A
A549
PBS
THBS2
B
H838 proliferation
NS
FoldChange (Normalized to PBS control)
1.0
0.5
0.0
PBS
THBS2
A549 proliferation
NS
FoldChange (Normalized to PBS control)
1.0
0.5
0.0
PBS
THBS2
C
LLc cell xenograft (C57BL/6 mice)
Tumor volume (mm³)
800
600
400
200
0
Ctrl
THBS2
****
0
5
10
15
Days after injection
Tumor Weight (mg)
600
400
200
0
***
Ctrl
THBS2
D
Control (PBS)
THBS2
100um

Figure S17. THBS2 promoted tumor micro-metastasis in A549 LUAD xenografts

A, Representative images (10x) of A549 transwell assays were shown.

B, The effect of THBS2 recombinant protein on the proliferation of LUAD cells (upper: H838; lower: A549). NS: not significant, by two-sided student`s t-test.

C, *In vivo* LUAD (mouse LLc cell line) xenografts showing the effect of THBS2 on subcutaneous tumor growth in immune-competent C57BL/6 mouse models. ****$p < 0.0001$ by two-way ANOVA test (tumor volume). ***$p < 0.001$ by two-sided student`s t-test.

D, Immunohistochemistry-based quantifications (related to the lower panel in Figure 7C); Qupath software (see the methods) was used to identify and quantify the KU80 positive cells. In B, another three representative IHC images showing the micro-metastasis to the lung in subcutaneous A549 LUAD xenografts. Human specific KU80 antibodies were used to detect human-derived A549 LUAD cells. Scale bar: 100 μm.
